# Modes of carbon fixation in an arsenic and CO_2_-rich shallow hydrothermal ecosystem

**DOI:** 10.1038/s41598-017-13910-2

**Published:** 2017-10-31

**Authors:** Nolwenn Callac, Nicole R. Posth, Jayne E. Rattray, Kweku K. Y. Yamoah, Alan Wiech, Magnus Ivarsson, Christoffer Hemmingsson, Stephanos P. Kilias, Ariadne Argyraki, Curt Broman, Henrik Skogby, Rienk H. Smittenberg, Ernest Chi Fru

**Affiliations:** 1 Stockholm University, Department of Geological Sciences and Bolin Centre for Climate Research, SE-106 91 Stockholm, Sweden; 2 Nordcee, Department of Biology-University of Southern Denmark Campusvej 55, 5230 Odense M, Denmark; 3 Department of Palaeobiology and Nordic Center for Earth Evolution, Swedish Museum of Natural History, Stockholm, Sweden; 4 Department of Geology and Geoenvironment, Section of Economic Geology and Geochemistry, National and Kapodistrian University of Athens, Panepistimiopolis, Zographou, 157 84 Athens, Greece; 5 Department of Geosciences, Swedish Museum of Natural History, Stockholm, Sweden; 6 Present Address: School of Earth and Ocean Sciences, Cardiff University, Park Place, Cardiff, CF10 3AT United Kingdom; 7 Department of Geosciences and Natural Resource Management – IGN University of Copenhagen, Øster Voldgade, 10 1350 København K Denmark

## Abstract

The seafloor sediments of Spathi Bay, Milos Island, Greece, are part of the largest arsenic-CO_2_-rich shallow submarine hydrothermal ecosystem on Earth. Here, white and brown deposits cap chemically distinct sediments with varying hydrothermal influence. All sediments contain abundant genes for autotrophic carbon fixation used in the Calvin-Benson-Bassham (CBB) and reverse tricaboxylic acid (rTCA) cycles. Both forms of RuBisCO, together with ATP citrate lyase genes in the rTCA cycle, increase with distance from the active hydrothermal centres and decrease with sediment depth. Clustering of RuBisCO Form II with a highly prevalent *Zetaproteobacteria* 16S rRNA gene density infers that iron-oxidizing bacteria contribute significantly to the sediment CBB cycle gene content. Three clusters form from different microbial guilds, each one encompassing one gene involved in CO_2_ fixation, aside from sulfate reduction. Our study suggests that the microbially mediated CBB cycle drives carbon fixation in the Spathi Bay sediments that are characterized by diffuse hydrothermal activity, high CO_2_, As emissions and chemically reduced fluids. This study highlights the breadth of conditions influencing the biogeochemistry in shallow CO_2_-rich hydrothermal systems and the importance of coupling highly specific process indicators to elucidate the complexity of carbon cycling in these ecosystems.

## Introduction

**Figure 1:**
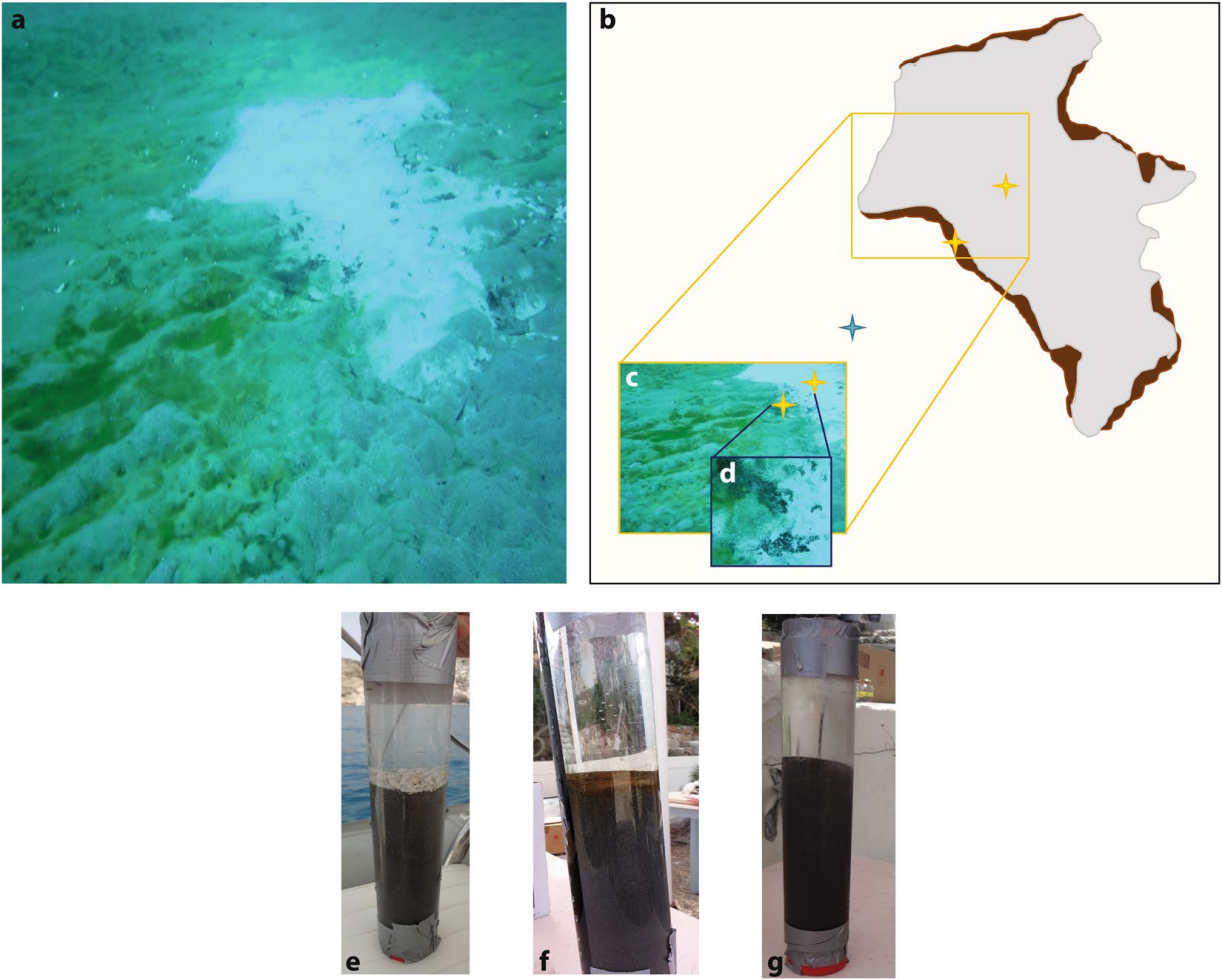
Sampling sites at Spathi Bay. (**a**) Photograph of the sampling site; (**b**) Schematic diagram illustrating where the sediment cores were sampled; (**c**) Photograph with yellow stars illustrating sample location in the white-capped and brown-capped sediments; (**d**) Photograph showing the junction between the white and the brown deposits. (**e**) Photograph of the push core collected in the white-capped sediment showing the white deposit at the interface between the sediment and the seawater; (**f**) Photograph of the push core collected in the brown-capped sediment showing the brown deposit at the interface between the sediment and the seawater; and (**g**) Photograph of the push core collected in the reference sediment where no deposits are visible at the interface between the sediment and the seawater. The photographs have been taken by Nolwenn Callac and Christoffer Hemmingsson.

Shallow submarine hydrothermal ecosystems occur in the photic zone at water depths less than 200 m below sea level^1,2^. Hydrothermal activity generally occurs at divergent and convergent boundaries such as subduction and seafloor spreading margins, fault zones, and in submarine serpentinization systems^1^. Marine shallow-water hydrothermal vents (SHV) in particular are often linked to arc volcanism, for instance on the Hellenic Volcanic Arc (HVA), Milos Islands (Greece), offshore of Kueishan Island (Taiwan), and Tutum Bay (Papua New Guinea)^2^. Because of the elevated temperature and extreme pH conditions, hydrothermal fluids are often rich in dissolved sulphur, iron, and other reducing inorganic chemical species (i.e., As, Cu, Pb, Hg) normally not present in appreciable amounts in low temperature marine settings^3,4^. Milos Island, an emergent volcano on the HVA in the Aegean Sea, bound by shallow hydrothermal submarine venting on its bays^4–6^ (Fig. 1), harbours one of the most As-enriched shallow submarine hydrothermal ecosystems on its shores^4,7,8^, characterised by vent fluids containing up to 3900 times more As than the overlying seawater^3^. On average, the hydrothermal fluids are up to 78.1 µM enriched in As at the 10 cm sediment depth^4^, with a maximum of 88 µM measured in endmember hydrothermal fluids at Spathi Bay^4^. Some seafloor hydrothermal areas along the coast in Palaeochori and Spathi Bays exhibit white, yellow-orange and brown deposits^4,5^. The white deposits are composed of amorphous silica and native sulphur^6^ and the yellow-orange deposits mostly consist of arsenic sulphide minerals^4,9^. The brown deposits contain manganese and iron oxides^10^. The active diffuse hydrothermal activity is characterized by gas venting and fluid flow through the white-capped sediments, and are composed of up to 90% CO_2_ and 2% CH_4_ (ref.^5^). The high CO_2_ emissions^5^ are a major potential source to fuel inorganic carbon fixation and drive *in situ* organic matter production in the sediment. While the rTCA cycle may drive carbon fixation in deep-sea hydrothermal systems rather than the Calvin Cycle^11,12^, the same cannot be assumed for shallow submarine settings. Indeed, there is a dearth of information on carbon fixation in shallow submarine settings, especially regarding the genetic quantification of specific taxa or functional genes, such as those involved in carbon fixation pathways. Also, owing to geothermal activity, exposure to light, and fluids enriched in As, shallow hydrothermal systems are generally considered extreme environments where both photosynthetic and chemosynthetic microbial processes^1,7^ occur simultaneously. Taken together, the Milos SHV offers an ideal opportunity to investigate the predominant modes of carbon fixation and carbon utilization in the extreme, As-influenced SHV sediments. Besides lending insight to the main drivers behind the biogeochemical cycling of carbon in such systems, a study of the shallow hydrothermal environment also helps to define how these settings differ from the more intensely studied deep-sea hydrothermal areas^11,12^.

Our aim here was therefore to investigate the mode of carbon fixation in the shallow submarine hydrothermal environment of Spathi Bay, Milos Island. Specifically, we focused on coastal, hydrothermally-affected and unaffected sediment as a function of depth and distance from the hydrothermal centre using a suite of biogeochemical techniques: microbial community profiling using quantitative polymerase chain reaction (qPCR) and lipid biomarker studies, as well as bulk geochemical analyses. We identified and quantified key carbon fixation genes from samples collected in three different habitats in Spathi Bay. Two habitats were capped either by white or brown deposits and one was a deposit-free sand reference (Fig. 1a–d). To evaluate a putative biological CO_2_ uptake, we also measured δ^13^C_org_, δ^13^C_DIC_ and performed lipid biomarker analyses to investigate biological actors other than microorganisms in the system.

Our results show a high spatial heterogeneity in carbon-fixing gene distribution related to the abundance of archaea, bacteria and *Zetaproteobacteria* 16S rRNA genes. Areas in the direct vicinity of the white (amorphous silica and sulphur-rich) and brown (manganese and iron oxide-rich) deposits have a lower abundance of genes involved in carbon fixation relative to the reference sediment. Irrespective of depth or habitat, qPCR analyses indicate that the Calvin Cycle is the main CO_2_ fixation pathway mediated by microorganisms in this shallow hydrothermal setting, with the predominance of *cbbm* genes quantified over *aclB* genes (rTCA cycle). The δ^13^C_org_ and δ^13^C_DIC_ composition of the sediment was found to be consistent with autotrophic carbon fixation processes. While CO_2_ consumption in the sediment is driven by both the hydrothermal CO_2_ gradient and microbial C-fixation, lipid biomarker analysis further revealed the influence of both plant and microbially derived organic matter in the sediments.

## Results

### Sediment mineralogy and porewater chemistry

X-ray diffraction (XRD) and Raman spectroscopy analysis revealed quartz, birnessite (Mn oxides), graphite and hematite (FeI(II)(oxyhydr)oxides) as ubiquitous minerals in the sediment, regardless of depth and hydrothermal influence (Tables S1 and S2). Fe-sulphides (pyrite, marcasite) were detected only in the white-capped sediments whereas goethite (Fe(III)(oxyhydr)oxides)) was found in both the brown-capped and reference sediments (see SI for detail results on mineralogy and porewater analyses).

**Figure 2:**
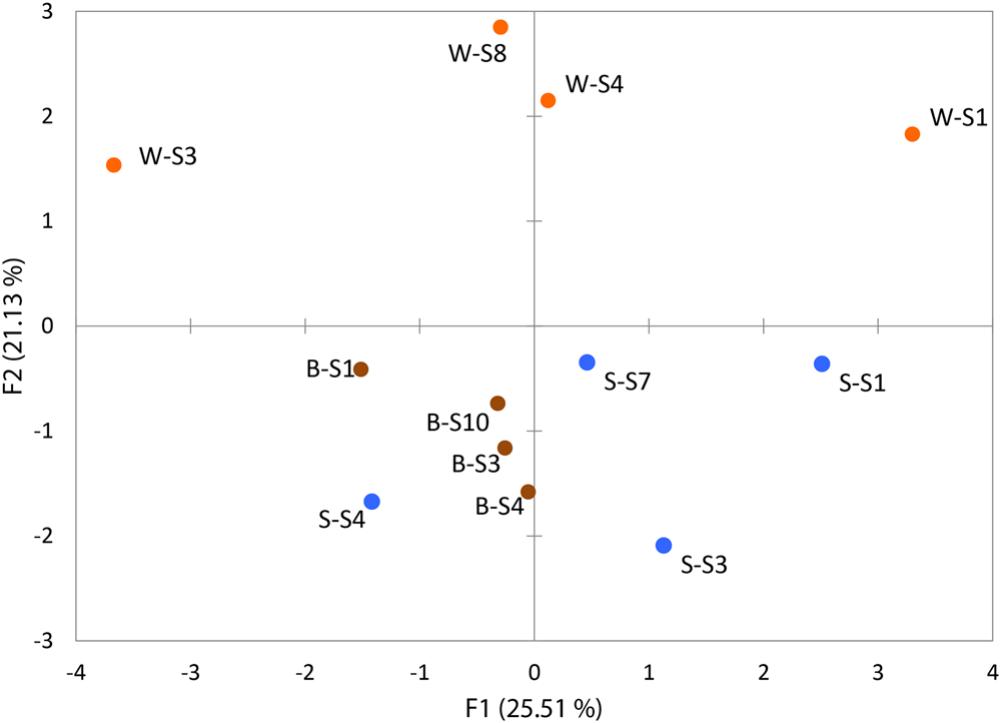
Principal Component Analysis (PCA) for Raman spectroscopy mineralogical data, showing same habitat samples clustering together, except for S–S4. The first letter: W, B or S refers to white-capped, brown-capped and reference sand sediments, respectively. S1 represents 0–2 cm, S3 = 3–4 cm, S4 = 6–8 cm, S7 = 12–14 cm, S8 = 14–16 cm and S10 = 18–20 cm.

The Raman data coupled to principal component analysis (PCA) indicates that the three habitats were distinct in mineralogical and chemical composition (Fig. 2). Additionally, the PCA analysis shows that the white-capped sediment was more mineralogically heterogeneous than the brown-capped and the reference sediments (Fig. 2). The porewater analysis revealed (SI results 1-Sediment mineralogy and porewater chemistry) that arsenic was generally abundant when detected (detection limit 2 ppb), especially in the white-capped sediments, and particularly at 14–16 cm depth where a maximum concentration of 42.7 ppm was measured (Table S3). Remarkably, porewaters were also depleted in phosphate, suggesting the ecosystem was phosphate-limited and the overlying seawater was generally depleted in elemental P relative to the sediments (Table S3).

### Carbon isotopic values in the core sediments

The bulk carbon isotopic composition δ^13^C_org_ of all white- and brown-capped sediments revealed small variations ranging between −18‰ and −20‰ (Fig. S1). The δ^13^C_DIC_ shows a narrow range of 2.1–4.7‰ in the reference sediments compared to ranges of 0.21–5.26‰ and 4.43–11.98‰ in the white- and brown-capped sediments, respectively (Fig. S1a–c). The resulting C-isotope fractionation expressed as the difference between DIC and POC, ∆^13^C = δ^13^C_DIC_ − δ^13^C_POC_, is approximately at 20–25‰ for these sediments. This range is consistent for the values expected for both CBB and rTCA dominated environments (see Canfield *et al*.^13^, for comparative values).

### Spatial distribution of genes quantified by qPCR

Quantification of targeted specific genes was performed by qPCR on two sediment cores per habitat (core 1 and 2), i.e., white-capped, brown-capped and reference sediment. The quantified genes were bacterial, archaeal and zetaproteobacterial 16S rRNA; *cbbL* and *cbbM* (RuBisCO form I and II, respectively); *aclB* (ATP citrate lyase β subunit in the rTCA cycle); *pmoA* (particulate methane monooxygenase); *mmoX* (soluble methane monooxygenase); *mcrA* (methyl coenzyme M reductase central enzyme) and *dsrA* (dissimilatory sulfite reductase α subunit, a key enzyme for sulphate-reduction) (see SI for qPCR bias).

Targeted genes were quantified in all cores and depths (Figs S2 and S3) with some exceptions, cited below, for which the genes were not detected during the qPCR analysis:

1. 16S rDNA specific to *Zetaproteobacteria*, were not detected after 4–6 cm depth in core 1 collected from the white-capped sediments.
2. *cbbM* genes were not detected between 2–4 cm and 4–6 cm in replicate cores sampled from the white-capped sediments.
3. *mmoX genes* for the aerobic methanotrophic *Alphaproteobacteria* were not detected at 6–8 cm and in the deepest section of core 1 collected from the sediments capped with the white deposits.
4. *pmoA* genes for the aerobic methanotrophic *Gammaproteobacteria* were not detected in the uppermost part of the reference material (core 1), in the white-capped sediments between 8–12 cm (core 1), and between 10–14 cm (core 2).
5. *mcrA* genes were not detected in the white capped sediments below 8 cm (core 1) and between 10–14 cm (core 2).

**Figure 3:**
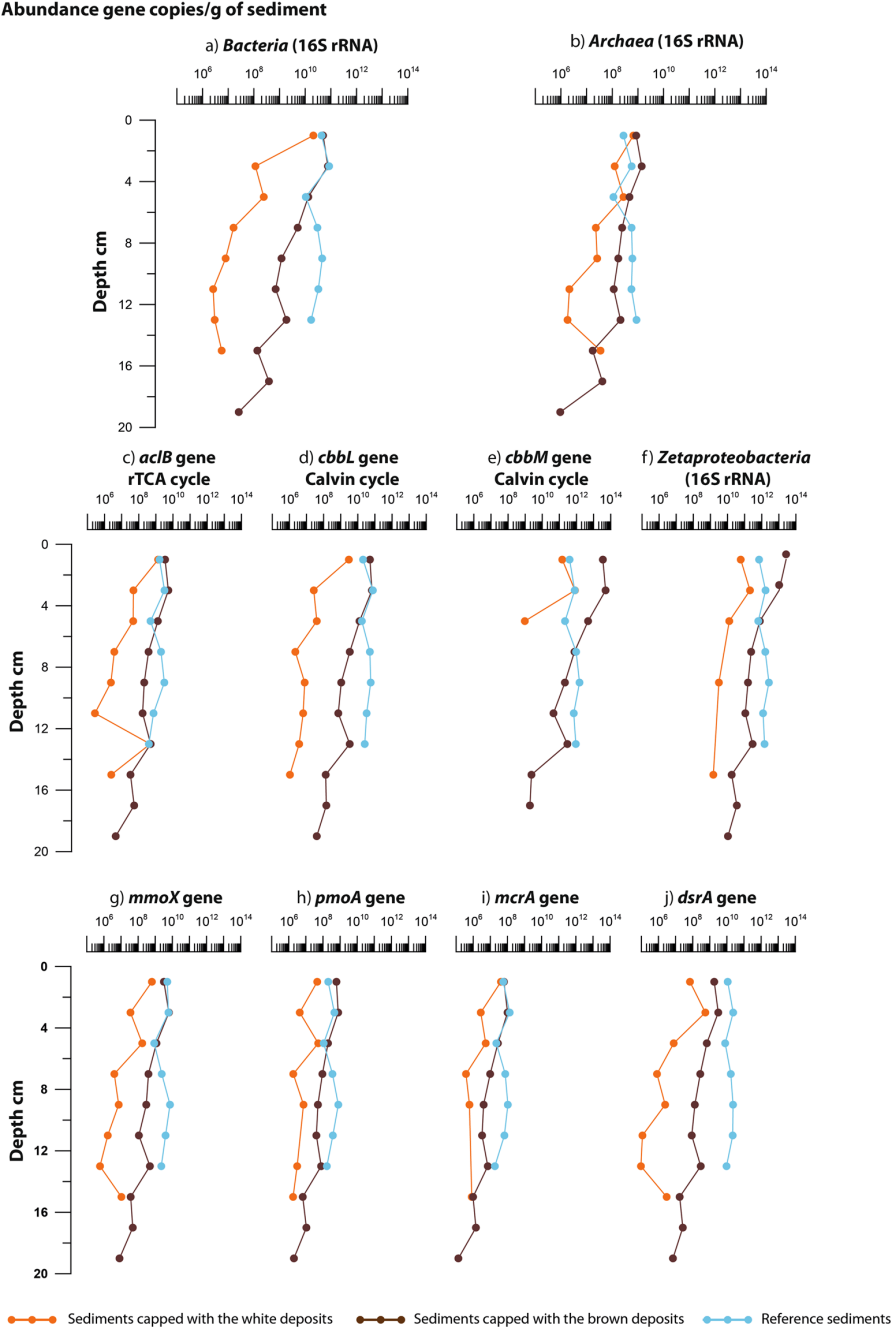
Average gene abundance for each section between the duplicate push cores collected in the same habitat, showing downcore gene distribution. The X-axis refers to the abundance of gene copies per gram of sediment for (**a**) Bacterial 16S rRNA gene; (**b**) Archaeal 16S rRNA gene; (**c**) *Zetaproteobacteria* 16S rRNA gene; (**d**) *aclB* gene; (**e**) the *cbbL* gene; (**f**) the *cbbM* gene; (**g**) the *mmoX* gene; (**h**) the *pmoA* gene; (**i**) the *mcrA* gene and (**j**) *dsrA* gene. The orange dots refer to qPCR data obtained in the white-capped sediment; brown dots to qPCR data obtained in the brown-capped sediment and blue dots to qPCR data from the reference sediment.

**Figure 4:**
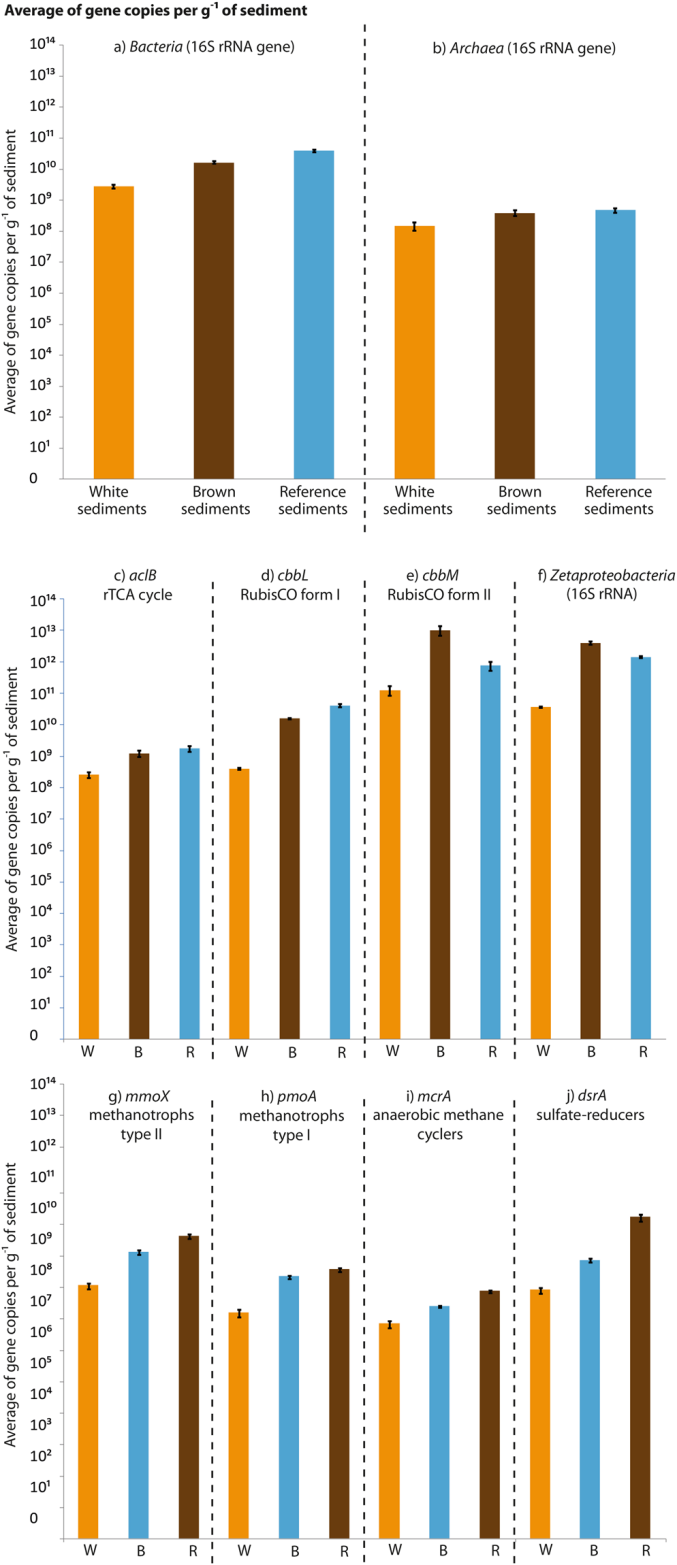
Calculated average gene abundance for whole cores. W, B and R represent white-capped, brown-capped sediments and reference sediments, respectively. The X-axis corresponds to the abundance of gene copies per gram of sediment for (**a**) Bacterial 16S rRNA gene; (**b**) Archaeal 16S rRNA gene; (**c**) *aclB* gene; (**d**) *cbbL* gene; (**e**) *cbbM* gene; (**f**) *Zetaproteobacteria* 16S rRNA genes; (**g**) *mmoX* gene; (**h**) *pmoA* gene; (**i**) *mcrA* gene and (**j**) *dsrA* gene. The bars show standard error between the duplicate whole cores of each habitat.

For all quantified genes, we observed numerical gene distribution maxima and minima in the reference and white-capped sediments, respectively. Generally, gene abundance decreased with depth at all sites (Figs 3, S2 and S3). In duplicate cores, differences in the same gene content were observed (Figs 3, S2 and S3), though the trends were similar between and within habitats. Common to all habitats, bacteria dominated over archaea in the top sediment layers. Archaeal abundance generally increased with depth and became more dominant in the white-capped sediments, making up ~86% of total prokaryotic 16S rRNA gene abundance at 15 cm (Fig. 3, Table S4). Bacterial 16S rRNA genes dominated prokaryotic 16S rRNA gene abundance in the reference sediments, making up 95–99% of total prokaryotic 16S rRNA gene quantification (Table S4). The *Zetaproteobacteria* community appeared to comprise a large part of the bacterial 16S rRNA gene pool, especially in the brown-capped sediments (Figs 3, 4 and S2) and their abundance was higher compared to total bacteria (Fig. 4), which can be explained by primer specificity (see SI for inherent biases in the qPCR method). The average gene abundance in replicate cores from the same habitat clearly show that the *Zetaproteobacteria* were an abundant taxon within the bacteria domain in the brown-capped sediments, at least in the first 6 cm (Figs 3 and S2).

In general, genes used for the Calvin-Benson-Bassham cycle dominated all samples, with a high majority analysed as genes associated with Form II RuBisCO (*cbbM*) and lower amounts associated with Form I (*cbbL)* (Figs 3, 4 and S2). The *cbbL* and *aclB* genes were similarly abundant in the white-capped sediments (Figs 4 and S2). The average gene abundance per habitat therefore highlights that *cbbM* genes were predominant in the shallow submarine sediments of Milos Island, especially in the brown-capped sediments (Figs 3 and 4).

### Spatial distribution of lipid biomarkers and lipid carbon isotopes

**Figure 5:**
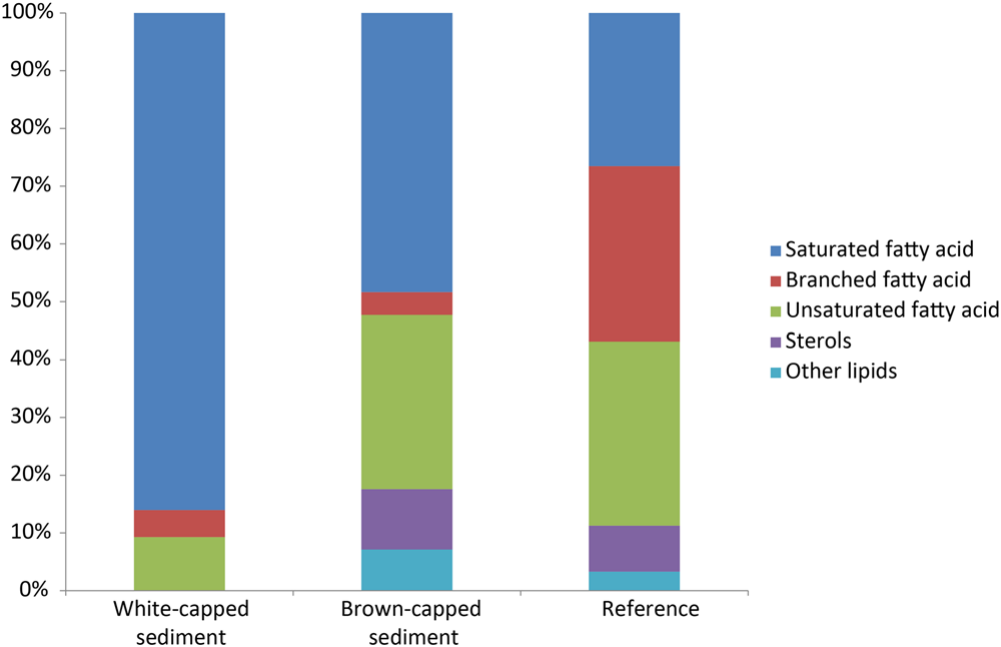
Histogram showing the relative abundance of lipid classes in the top section (0 to 2 cm) for the three habitats. The relative abundance of lipid classes has been calculated using their respective concentrations in the top section (0 to 2 cm) for the three habitats.

Lipids specific to bacteria, the *iso*-C15:0 and *anteiso*-C15:0 fatty acids, were more distributed near the surface of the brown-capped and the reference sediment and less abundant in the white–capped sediments (Table S5). Sterols, especially the C29 4-desmethyl sterols typically produced by eukaryotes^14^, were found in all three habitats, with a predominance in the brown-capped and sand reference sediment. β-sitosterol was the most abundant sterol in the upper sediment layer at all sites. Lipids of non-determined origin (i.e. potentially biosynthesised by both bacteria and eukarya) had the highest distribution, which generally decreased downcore at each site. Saturated fatty acids were found at all sites and at all depths, but the unsaturated C16:1 cis-9 fatty acid was only present in surface layers at the white and brown-capped sites. Both saturated and unsaturated fatty acids showed an increasing distribution trend across the different habitats, i.e. going from the white to the brown-capped to the reference sediments (Fig. 1b–c). To compare the total lipid distribution at each site, all data were sorted into respective lipid classes (Fig. 5). The highest diversity of lipid class was observed in the brown-capped and the reference sediments and the lowest diversity in the white-capped sediment.

Carbon isotopic contents of the bacteria-specific *anteiso*-C15:0 fatty acid and *iso*-C15:0 fatty acid in the reference sediment were −19.7‰ and −22.6‰, respectively (Table 1). In comparison, the more generic C16:1 cis-9 and C16:0 fatty acids had respective isotopic compositions of −15.7‰ and −25.5‰, −29.7‰ and −25.5‰ and −19.4‰ and −19.9‰, for the white–capped, brown–capped and reference sediments, respectively. The C18:0 fatty acid was below detection limit in the white-capped sediments, but had an isotopic composition of −31.6‰ and −25.7‰ in the brown-capped sediments, and the reference sediments, respectively.

**Table 1:** Lipid carbon isotopic values (‰ *δ* ^13^ *C vs VPDB*) in the top surface (section 0 to 2 cm depth). In the three sites.

Lipids	White capped sediment	Brown capped sediment	Sand sediment (Reference)
**Bacterially derived lipids**			
*anti iso*C15:0 fatty acid	<lod	<lod	−19.7‰
*iso*C15:0 fatty acid	<lod	<lod	−22.6‰
**Lipids of non-specific origin**			
C16:1 cis-9 fatty acid	−15.7‰	−29.5‰	−19.4‰
C16:0 fatty acid	−25.5‰	−29.6‰	−19.9‰
C18:0 fatty acid	<lod	−31.6‰	−25.7‰

lod limit of detection.

### Statistical analysis

The MANOVA (Multivariate analysis of variance) test indicated that habitat (e.g., white-, brown-capped and reference sediments) generally played an important role in the amount of gene quantified by qPCR (*P* < *0.005*) (Table S6). Indeed, besides the archaeal 16S rRNA and *aclB* gene abundance in the brown-capped and reference sediments and the *dsrA* gene quantification in the white and the brown-capped sediments (Table S6), which showed no statistical difference, all the other qPCR in the three habitats were significantly different. Using the MANOVA test, no statistically supported differences regarding specific gene abundance were found across individual, replicate push cores of the white-capped sediments (Table S7). The same test on replicate brown-capped and reference sediments, however, resolved intra-site variability (Tables S7 and S8) (SI 2-Statistical analysis). Significant correlations were also found between genetic quantification and several depths and are detailed in the SI (SI 2-Statistical analysis and Table S8).

**Figure 6:**
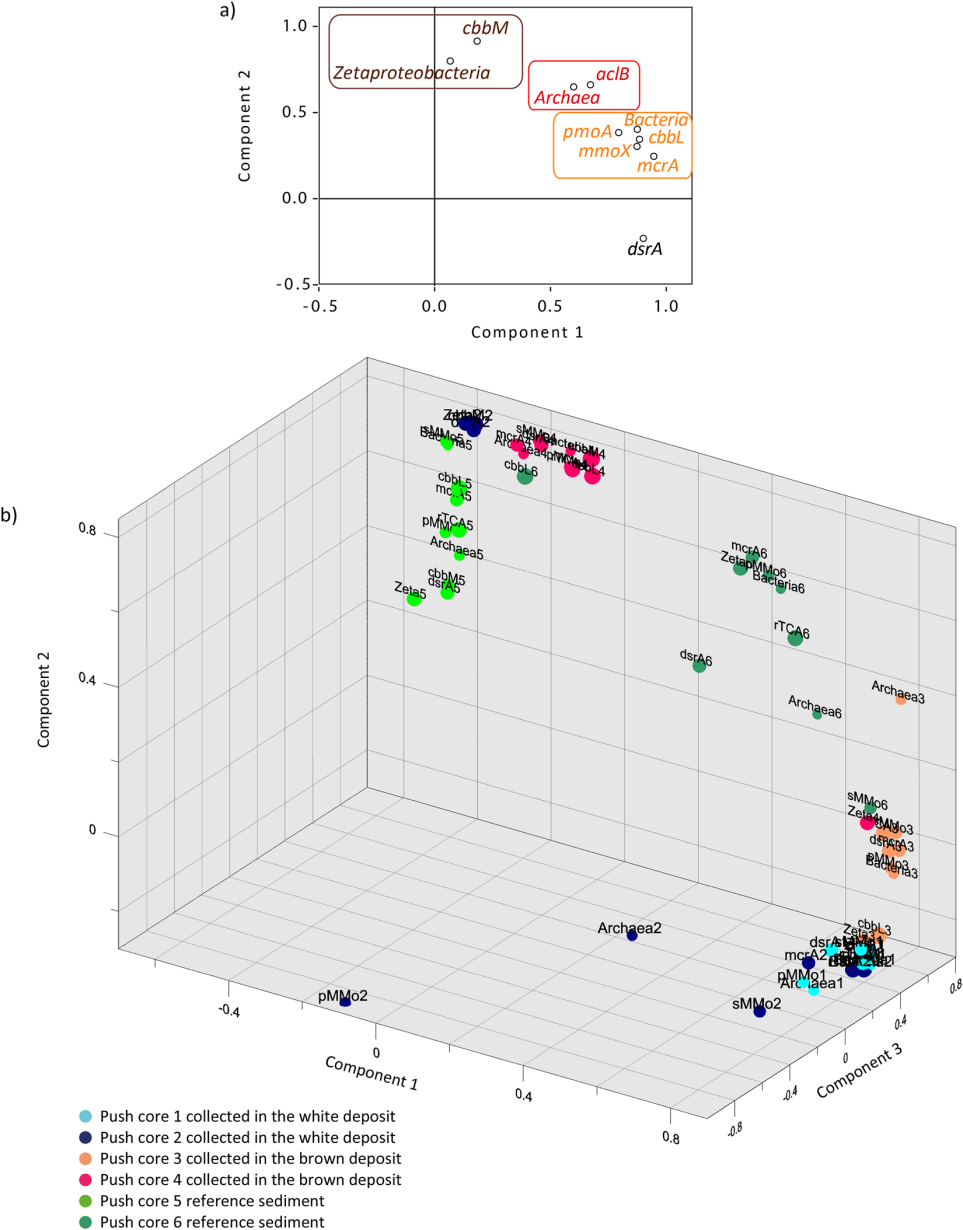
Factor analysis diagrams. (**a**) Factor analysis diagram showing the relationships between genes quantified by qPCR irrespective of the habitat. Cluster 1, 2 and 3 are represented by brown, red and orange, respectively. Bacteria refers to quantified bacterial 16S rRNA genes; Archaea to archeal 16S rRNA genes; and Zetaproteobacteria to zetaproteobacterial 16S rRNA genes; (**b**) 3-D Factor analysis diagram showing the relationships between each push core and genes quantified by qPCR. Bacteria refers to quantified bacterial 16S rRNA genes; Archaea to archeal 16S rRNA genes; Zetaproteobacteria to zetaproteobacterial 16S rRNA genes; rTCA to *aclb* genes; sMMO to *mmoX* genes; and pMMO to *pmoA* genes.

The factor analysis calculated without considering core origin, replicates and depth, emphasize that the genes were grouped in three main clusters and that the *dsrA* gene are independent of others (Fig. 6a). Cluster 1 encompassed the *Zetaproteobacteria* and the *cbbM* genes (Fig. 6a). Cluster 2 grouped the archaeal 16S rRNA gene abundance with *aclB* genes in the rTCA cycle, while cluster 3 included bacteria 16S rRNA gene abundance, *mmoX*, *pmoA*, *mcrA* and *cbbL* genes (Fig. 6a). 3D-factor analysis revealed variability between replicates from the same habitat, meaning that independent push cores behave as sub-habitats within habitats (Fig. 6b).

## Discussion

Spathi Bay is a shallow submarine environment influenced by high arsenic concentrations (average 78 µM As in 10 cm sediment depth and up to 88 µM in endmember hydrothermal fluids). Gaseous emissions from the white-capped sediments at this site contain up to 90% CO_2_ and 2% CH_4_ (ref.^8^)_,_ available to chemoautotrophs. Regardless of depth or habitat, qPCR analysis suggests that the main CO_2_ fixation pathway is the microbially-mediated Calvin Cycle (Figs 3, 4 and S2). RuBisCO form II, quantified *via cbbM* genes and generally found in CO_2_-rich environments^15^, is quantitatively predominant in the Spathi Bay sediments as well (Figs 3, 4 and S2) (See SI for the six main pathways of the inorganic carbon fixation). This predominance of genes associated with the Calvin cycle in shallow hydrothermal sediments differs from the quantitative predominance of the rTCA cycle reported for some deep-sea hydrothermal settings^11^. Metagenomic analyses performed on shallow hydrothermal sites located at Kueishantao, Taiwan, and the Kolumbo site of the HVA to which Milos belongs, however, have found evidence of both rTCA and CBB-driven carbon fixation^16,17^. The apparent dominance of the CBB cycle as the main CO_2_-fixation pathway found here at Spathi Bay may be due to the large amount of CO_2_ in the gaseous emissions in the white-capped sediment as RuBisCO form II is most effective in CO_2_-rich environments^15^. Furthermore, CO_2_ gradients in focused fluid emissions areas, such as chimney-dominated systems, may be more established than in the diffusive sediment of Spathi Bay. The lack of strong gradients in Spathi bay sediments may explain the lack of a distinct rTCA to CBB transition related to the changing dominance of *Epsilon-* to *Gamma-proteobacteria* with distance from the vent, as has previously been suggested for SHV fields^2,18^.

Microbial abundance and diversity differ greatly in the white sediment, brown sediment and reference sediments studied herein (Fig. 1e–g). As in past microbial abundance studies at Milos Island, a high microbial abundance was found in the reference sediment^18^, which highlights the influence of temperature as well as As and other reduced species on microbial distribution. The mineral zonation in the sediment cores is clear from Principle component analysis (PCA) of the sediment mineralogy (Fig. 2) and shows key differences in the three habitats studied. The concentrations of Ca, K and Mn were higher in porewater collected from the white- and brown-capped deposits and lower in the reference sediments (Table S3). This points to a higher hydrothermal contribution in the capped sediment than in the deposit-free sediment. Furthermore, the concentration gradient of Ca, K and Mn implies that the white-capped sediments are more hydrothermally-affected than the brown-capped and reference sediments; the latter being the least affected as shown from a previous study focusing on the hydrothermal sediments of the Guaymas basin^19^ (Table S3).

Dissolved iron concentrations were low in the porewater of all hydrothermally-affected sediments. In light of the sediment mineralogy, this might indicate Fe removal in the form of Fe-sulphides (pyrite, marcasite) in the white-capped sediments (Table S2) and mainly as Fe(III)(oxyhydr)oxides (hematite, goethite) in the brown-capped and reference sediments (Table S2). Furthermore, the white and the brown deposits may act as scavenging caps, regulating porewater trace metal and metalloid content. Indeed, this may especially be the case with the arsenic content in the sediments, as it is less abundant in surface sediment porewater than at depth (samples W2-S1 and B2-S2 Table S3). Indeed, the top surface of the white-capped and brown capped-sediments are composed of sulphide minerals and some Mn oxides, hematite (Fe_2_O_3_) and goethite (FeO(OH)), which are known to strongly regulate porewater trace metals and metalloid concentration^20^. Across sediments, the arsenic concentration in the porewater decreases from high concentration in the white-capped sediments to low concentration in the reference sediment (Table S3). In the hydrothermally-affected white- and brown-capped (samples BB1 and BB2 Table S3) sediments, the arsenic concentration increases with depth (Table S3). Similar observations regarding the arsenic behaviour in the sediments were made in a previous study in Spathi Bay, in which higher arsenic concentrations were detected in the hydrothermally-affected sediment covered by an orange patch than in the surrounding sediment covered by a white patch^4^. As in our study Price *et al*. (2013) also show, that the arsenic concentration increases with depth in the sediment covered by a white patch^4^. Arsenic appears not to be a major selector of major carbon fixation genes that dominate along the sampled transect, indicated by the homogeneity of similar dominant carbon fixation genes in the different habitats, despite the heterogeneity of arsenic concentrations. However, the systematic increase in the most abundant carbon fixation pathway genes from the hydrothermal centres to the reference sediments, while arsenic content declines simultaneously in the same direction, suggests that arsenic may be a contributing factor to the decrease in hostile habitable conditions in this direction. This proposition is supported by our observation that the hydrothermal centres are generally the most hostile habitats in this environment.

Arsenic is a toxic metalloid known to produce cell impairment by compromising for example phosphate metabolism and the cell’s protein function machinery. By acting as a phosphate analogue in various cell compounds^21^ [and references therein], arsenic species prevent DNA replication, ATP formation or phospholipids and phosphoprotein synthesis. The arsenic gradient in the sediment, in addition to the hydrothermal activity, therefore, offers one possible explanation for why the white-capped sediment is less inhabited than the brown-capped sediment (Figs 3, 4, S2 and S3). In turn, this also explains why the brown-capped sediment is less inhabited than the reference sediment (Figs 3, 4, S2 and S3). Consequently, the upper sediment seems to be less hostile to microbial life than deeper sediment. This may be especially true for the hydrothermally affected sediments where habitability increases from the white-capped to the brown-capped to the reference sediments (Figs 3, 4, S2 and S3). Thus, overall habitability at least in the white-capped and brown-capped sediments is suppressed, allowing only specialists and extremophiles to thrive in that environment.

MANOVA (Tables S6, 7 and 8) and 3D factor analysis (Fig. 6b) show that gene abundance quantified by qPCR is dependent on the habitat, push core, and depth. This dependence on location of gene abundance measured by qPCR could explain why the bacterial and archaeal 16S rRNA gene abundance show a decreasing trend with depth in all cores (Figs 3, S2 and Table S5). Bacterial 16S rRNA genes were more abundant in the brown-capped and reference sediments, whereas the white-capped sediments generally show higher archaeal 16S rRNA gene content (Figs 3, S2 and Table S4). Similar to the total microbial 16S rRNA gene abundance (bacteria and archaea combined) (Fig. 3), lipid concentrations increase in the sediment with distance from hydrothermal influence (Table S5). The significance of the hydrothermal zonation (from white- to brown-capped sediment, to the reference) was further supported by *iso* C15:0 and *anteiso* C15:0 fatty acids content, which are common biomarkers for the *Bacillus* lineage^22,23^. Indeed, the C15:0 *iso*/*anteiso* ratio is known to change in response to temperature variations^22,23^. A distinct increase in the C15:0 *iso*/*anteiso* ratio from 1.15, 1.50 to 3.92, was observed from the reference to the brown-capped and white-capped sediments, respectively. This change in the C15:0 *iso*/*anteiso* ratio also highlights the change in hydrothermal activity and in temperature from high in the white-capped sediment; to low in the brown-capped deposits, and insignificant values in the reference sediment. A study for the obligate thermophile^22^, *Bacillus megaterium* cultured at 70 °C reports a ratio of 3.95, implying that at similar temperature, thermophilic growth supports the biosynthesis of the *iso* C15:0 and *anteiso* C15:0 fatty acids in the white-capped sediment. In contrast, the lower ratios in the brown-capped and reference sediments correspond to temperatures of 40 °C and 30 °C, respectively, which are temperatures commonly inhabited by mesophiles. It is thought that the sensitive change of *iso* C15:0 synthesis in *Bacillus sp*. is directly correlated to its survival in extreme environments^23^. In addition, to corroborate the estimated temperature in the brown-capped sediment, several *Bacillus* strains have been isolated at 35 °C from the top section (0 to 4 cm depth) (data not schown). We hypothesise that the hydrothermal activity and gradient directly affects microbial abundance in the surface sediment as has been seen by previous studies^2,18^. This would also explain why the microbial abundance of the 16S rRNA genes and the bacterially derived lipids are most abundant in surface sediments compared to deeper samples, and in the reference sediments relative to the hydrothermally affected sediments.

Statistical analysis (Fig. 6a) revealed strong correlation between different guilds of microorganisms, highlighting preferential prokaryotic associations. Thus, factor analyses (Fig. 6a) indicate an important correlation between the chemoautotrophic *Zetaproteobacteria* and RuBisCO form II (*cbbM)* genes, which is consistent with studies in Hawaiian and Greek hydrothermal settings^17,24^. Moreover, while both *cbbL* and *cbbM* genes have been detected in the genome of *Marinoprofundus ferooxydans* ^25^, factor analysis revealed a cluster for only the *cbbM* gene with the *Zetaproteobacteria* in the Spathi samples. This suggests that the *Zetaproteobacteria* contribute significantly to the abundance of the *cbbM* genes in Milos’s sediments probably due to the high CO_2_ content of this environment. The factor analysis also grouped together the archaeal 16S rRNA genes with the *aclB* gene, suggesting that archaea likely contribute the most to the *aclB* gene content (Fig. 6a). This correlation is interesting because in deep-sea hydrothermal systems, the *aclB* gene is detected mainly in *Epsilonproteobacteria* and *Aquificales* within the bacteria and in *Thermoproteacea* within the archaea^11,26^. The factor analysis further revealed a cluster consisting of bacterial 16S rRNA, *cbbL* (RuBisCO form I), *mmoX*, *pmoA* (bacterial aerobic methanotrophy) and *mcrA* (archaeal anaerobic methane cycling) genes (Fig. 6a). RuBisCO form I is present in the *Proteoabacteria* and *Cyanobacteria* ^27^. However, some methanotrophs and archaea, including *Methanocaldococcus jannaschii* ^28^, *Methanosarcina acetivorans* ^29^ and *Methanosarcina mazei* ^30^, are also known to use RuBisCO form I^31^. Metagenomics data we have generated for a different study of the same site suggest the sediments are poor in genes encoding photosystems I and II (data not shown). Furthermore, the entire metagenomics dataset, comprising ∼4000 assembled functional genes, indicates that oxygenic photosynthesis is not an important process in carbon fixation in this environment (data not shown).

In the white-capped sediments and in the reference sediments, a significant correlation exists between the *mcrA* and *dsrA* gene (methyl coenzyme M reductase central enzyme for methanogenesis or anaerobic methane oxidation^32,33^ and dissimilatory sulphite reductase α subunit, a key enzyme for sulphate-reduction^34^, respectively). This correlation, however, was low in the brown-capped sediments. These relationships highlight that some form of anaerobic methanotrophy coupled to sulphate reduction may be expressed in the white-capped sediments and in the reference sediments, although the cluster analysis did not support this conclusion. Further evidence for microbial sulphate-reduction is provided by the prevalence of Fe sulphide as pyrite and marcasite minerals mainly in the white-capped sediments (Table S2). However, whether anaerobic methane oxidation is linked to sulphate reduction or to metal-reduction remains to be determined by gene expression studies or FISH analysis. Our study also suggests that aerobic methanotrophs capable of utilizing sMMO or pMMO enzymes may be widespread in marine and hydrothermal environments, though the pathway dependent on the sMMO enzyme has typically escaped cultivation. The genetic association between the *pmoA*, *mmoX* (aerobic methane cycling) and the *mcrA* (anaerobic methane cycling) genes highlight the close connection between the methane producers and consumers, as well as the possible involvement of anaerobic bacteria methane oxidation *via* nitrite reduction by internally producing O_2_ in anoxic environments^35^.

The sulphate reduction *dsrA* gene is the sole gene not clustered with others and it also does not group with any autotrophic carbon fixation pathway genes (Fig. 6a). The reasons for this are unclear, but may be related to the fact that most sulphate-reducing bacteria isolates are chemo-organotrophs^36^. For instance, Chemo-organotrophy by the sulphate-reducing microorganisms is responsible for recycling up to 50% of all organic carbon in anoxic marine sediments^37^. Interestingly, the presence of sterols suggests that a portion (around 40 to 50%) of the sedimentary organic matter is plant-derived. Indeed, the ratio of cholesterol to β-sitosterol, being 0.54, 0.40 and 0.52 in the top layers of the white, brown and reference sediments, respectively, indicates that some organic matter in the marine sediments may be of higher plant origin (Fig. S4). Cholesterol can be regarded as a zoo- and phytoplankton biomarker^38^ whereas β-sitosterol is primarily found in higher plants^39^. Based on observations surrounding the sampling site in Spathi Bay, previous work on Palaeochori Bay, and the sterol and fatty acid composition of seagrass origin, we hypothesize that β-sitosterol probably originates from the seagrass observed living in the bay (Fig. S4)^40,41^. Seagrass can exhibit relatively enriched carbon isotope values of around −10‰ (ref.^42,43^) which could explain some of the more enriched C16:0 fatty acid δ^13^C values. However, microorganisms using the rTCA cycle may also produce fatty acids of similar isotopic composition.

A recent study from the SHV fields of Dominica Island that support a prominent *Zetaproterobacteria* population similar to the one suggested for the Spathi Bay sediments by our study, proposes that chemoautotrophy may account for up to 65% of total organic carbon production in SHV fields compared to photosynthetic processes^44^. Relative importance of chemoautotrohy for primary production in a light exposed marine shallow hydrothermal system further revealed that chemoautotrophic processes were promoted by microorganisms containing *anteiso*-C_15:0_, C_15:0_, and *iso*-C_16:0_ fatty acids. Related bacteria-specific *anteiso*-C_15:0_ and *iso*-C_15:0_ fatty acids identified in the sediments at Spathi Bay show typical CBB cycle δ^13^C signatures of up to −22.6‰. The values fall within the range of our bulk sediment δ^13^C_org_ measurements, indicating that chemoautotrophs that produce the *anteiso*-C_15:0_ and C_15:0_ fatty acids, together with microbes with the more generic fatty acids that expressed δ^13^C values of −19.4 to −19.9‰, contribute the most to carbon fixation via the CBB cycle in the Spathi Bay sediments. Given the bulk range for δ^13^C_org_ in the sediments, we suggest that lineages that produce predominantly fatty acids with δ^13^C values lower than −20‰, contribute the least to sediment organic carbon production. Moreover, because seagrass is expected to enrich heavy ^13^C in plant fatty acids by a factor of two relative to the bulk sediment δ^13^C values recorded in this study^44,45^, we caution that while good preservation of plant organic matter is likely taking place in the sediment, organic matter contribution by plants to the total sediment production pool is likely negligible. The δ^13^C values of the ubiquitous C_16:1_ cis-9 fatty acids in the white– and brown–capped sediments, show a significant difference of 14‰. The diffuse CO_2_ emissions in the white and brown sediments alone insufficiently explain this disparity. The δ^13^C values of the C_16:1_ cis-9 fatty acids are more depleted in the brown-capped sediments than the reference sediment, which is expected to have the lowest CO_2_ concentration. The more likely explanation for this discrepancy is the influence of *in situ* carbon fixation by organisms using the rTCA cycle.

*In situ* production of fresh organic compounds therefore, likely provides available substrates for the heterotrophic and chemotrophic microbial community, such as sulphate-reducers. The Spathi Bay sediments contain the genetic capacity to enable both autotrophy *via* the CBB and rTCA cycles and heterotrophy with sulphate-reducers, which are not grouped with any of the autotrophic targeted genes (Fig. 6a). Evidence for plant fatty acids in the sediments might be argued to explain the high *cbbL* gene content in all the habitats, because of the possibility of detecting and quantifying eukaryotic *cbbL/rbcL* genes during qPCR analysis^45^. However, the similarity observed between bulk sediment δ^13^C_org_ values and bacterial-specific fatty acids signatures in the sediments, hint that plant contribution to the total organic matter pool is probably negligible, However, all together, the data support our finding that a microbially-mediated CBB cycle is the key CO_2_ fixation pathway in Spathi Bay driven by hydrothermal activity. This finding differs from the deep-sea hydrothermal setting where the rTCA cycle is found to be the prevalent CO_2_ fixation pathway.

## Conclusions

Our gene abundance quantified by qPCR data shows that hydrothermally active sediments, composed of white-capped sediments surrounded by brown-capped sediments, are marked by low gene abundance compared to areas not affected by direct hydrothermal activity (reference sediments). This, in addition to the fatty acid C15:0 *iso*/*anteiso* ratio, highlights a habitability gradient driven by hydrothermal activity.

The data also indicate that hydrothermal active and non-active sediments of Spathi Bay form a complex system where the carbon cycle is driven by both biotic and abiotic reactions, as well as by autotrophic and heterotrophic metabolic activities. The CBB cycle, and especially RuBisCO form II (encoded by the *cbbM* gene), is the main driver of CO_2_ fixation within the microbial community in the sediments. This seems to be especially true for the *Zetaproteobacteria* for which the specific 16S rRNA gene abundance coincides with the *cbbM* gene content. The qPCR data show that different microbial guilds are grouped into three clusters, each one encompassing one gene involved in CO_2_ fixation (*cbbL*, *cbM* or *aclB*) with the exception of the sulphate-reducers. The sulphate-reducers are not associated with any other quantified genes, highlighting their role in the heterotrophic part of the carbon cycle. Sedimentary organic carbon appears not only to be derived from *in situ* production, but also from extraneous sources, for instance seagrass fields further away. Our study suggests that the microbially-mediated CBB cycle drives carbon fixation in the Spathi Bay sediments of the Milos SHV characterized by diffuse, high CO_2_ emissions and high As and reduced species exudation. This study highlights the breadth of conditions influencing the biogeochemistry of SHVs and the importance of coupling different methods of highly specific process indicators to elucidate the complexity of the carbon cycle in shallow CO_2_-rich hydrothermal systems.

## Material and Methods

### Sample collection

Shallow water hydrothermal sediments were collected at Spathi Bay, 36°40′N; 24°31′E (Fig. 1a–g), southeast of Milos Island, at 12.5 m below sea level. As stated above and shown in Fig. 1b, the site is characterised by the emission of mainly CO_2_ and characteristic zones of white- and brown-capped seafloor deposits surrounding the vent area^5,46^. The sampled site had a white-capped sediment-seawater interface extending 8 m long by 3–5 m wide, rimmed by a brown-capped deposit (Fig. 1a–g). Local spots of hydrothermal emission are characterized by gas bubbles located within the white-capped deposits (Fig. S3). Large areas of sandy sediments, not visibly affected by direct hydrothermal activity (i.e., lacking gas emission and surface-capped coloured precipitates) extend from the rim of the brown-capped deposits outward. Sampling was therefore performed along a transect extending from the sandy outskirt through the brown- to white-capped deposit, to capture the metabolic potential of these three distinct seafloor habitats. The unconsolidated sediments were collected by SCUBA diving using polycarbonates push core tubes and capped underwater with rubber stoppers. Duplicate cores, from each habitat, were collected for geomicrobiological survey (geomicrobiology cores 1 and 2) and for the chemistry (chemistry cores 1 and 2), with polycarbonate tubes containing pre-drilled holes for the extraction of porewater, using rhizones, for geochemical and isotopic analysis. Cores for geomicrobiological analyses were subsampled under a N_2_ gas atmosphere in the field using an anaerobic glove bag (Glove Bag Inflatable Glove Chamber, Cole-Parmer). The sediment cores were sliced every 2 cm. Push core 1 collected in the brown-capped deposit was sliced every 4 cm using a disposable sterile plastic spatula and then stored on dry ice. Each section was divided into two: one for genetic survey and the other for corresponding geochemistry. These samples were stored in 50 ml Falcon tubes on dry ice, until subsequent storage at −80 °C in the laboratory. Porewater was extracted from the chemistry replicate cores using rhizones connected to depressurized exetainer^®^ bottles. Exetainers for DIC concentration and isotopic analysis were stored upside down at 4 °C until analysis. After porewater collection, one push core per habitat was transferred into the glove bag and sliced aseptically every 2 cm for further bulk δ^13^C isotopic analysis. Samples were placed in 50 ml Falcon tubes and stored under N_2_ in an anoxic bag, frozen and shipped on dried ice. At the laboratory, they were frozen at −20 °C.

### Sediment mineralogy and porewater chemistry

Powder X-Ray Diffraction (PXRD), using a PANalytical X’pert diffractometer equipped with an X’celerator silicon-strip detector was used to analyze the mineralogical composition of sediments. The instrument was run at 45 kV and 40 mA using Ni-filtered Cu-Kα radiation (*λ* = 1.5406 Å). Samples were run between 5–80° (2θ) in step sizes of 0.017° in continuous scanning mode while rotating samples. The top, middle and bottom portions of cores were used to define depth mineralogical-profiles. Samples were dried at 60 °C overnight and powdered in an agate mortar before analysis.

Raman spectroscopy was done with a confocal laser Raman spectrometer (LabRAM HR 800; Horiba Jobin Yvon, Villeneuve d’Ascq, France), equipped with a multichannel air-cooled charge-coupled device detector as previously described in Chi Fru *et al*.^47^. Briefly, the acquisition was obtained with an 1800 lines/mm grating and the excitation was done with an Ar-ion laser (514 nm) source. A confocal Olympus BX41 microscope was combined to the instrument. The laser beam, with a power at the sample surface at 8 mW, was focused through an 80x objective with a long-working distance of 8mm. The analysed spot size was around 1 µm. The spectral resolution was ~0.4 per cm/pixel and the accuracy of the instrument was controlled by repeated use of a silicon wafer calibration standard. The data collection and spectral baseline correction were done with the LabSpec 5 software.

Porewater concentrations of major and trace elements were measured using Inductively Coupled Plasma-Atomic Emission Spectrophotometry (ICP-AES, Varian Vista AX) at Stockholm University (details are given in SI methods).

### Sediment carbon isotope analysis for the assessment of δ^13^C_DIC_ and δ^13^C_org_

Carbon isotopic analysis: δ^13^C_DIC (Dissolved Inorganic Carbon)_ and δ^13^C_org (organic carbon)_, were analysed respectively using a GasBench II coupled to a Delta V Plus mass spectrometer (ThermoFinnigan) following the set-up described in Torres *et al*.^48^ with the exception that a chilled autosampler was not used and a EA-IRMS (Thermo-Delta V Advantage Isotope Ratio MS, EA Flash 2000 Organic elemental analyzer isotope ratio mass spectrometry, SDU Odense). Details about the procedure are in the SI (SI methods).

### DNA extraction and Quantitative polymerase chain reaction (qPCR)

DNA extraction was performed in duplicates from ~0.25 g of sediment, using the Mo Bio PowerSoil DNA kit (Carlsbad, CA) according to the manufacturer’s instructions. The 16S rRNA gene abundance of archaea, bacteria, *Zetaproteobacteria, cbbL, cbbM*, *aclB*, *pmoA*, *mmoX*, mcrA and *dsrA* genes (Table S9), were quantified by qPCR. Amplifications were done in 96 well qPCR plates using a CFX96 Touch™ Real-Time PCR Detection System (C1000 Touch™ Thermal, Cycler, Bio-Rad) Instrument and supplied software. The reactions were carried out in final volumes of 25 µL, using the SsoAdvanced^TM^ Universal SYBR^®^ Green Supermix (Bio-Rad), following the manufacturer’s recommendations. Samples contained 5 µL of DNA (1 ng.µL^−1^), specific primer set at appropriate concentrations and annealing temperatures (Table S9), in 35 cycle reactions. Standard curves were calibrated using ten-fold dilutions from pure cultures representing organisms carrying targeted genes (Table S9). The qPCR detection of 16S rRNA and specific genes in samples and in standards was run in triplicates, alongside negative controls to rule out laboratory contamination. The total gene copy numbers per gram of sediment was calculated from the triplicate sample averages as previously described^49^. Taxa 16S rRNA gene abundance was estimated according to the average of 1.86 and 4.1 for 16S rRNA genes per cell of archaea and bacteria, respectively^50^ and 1.5 for the *Zetaproteobacteria* ^51^), two copies of the pMMO-encoding *pmoA* protein per cell^52^, one for the sulphate-reducing bacteria *dsrA* gene^53^. From the sparse data available for the number of specific genes of *mcrA*, *mmoX*, *cbbL*, *cbbM*, *aclB* per genome, especially in prokaryotes inhabiting the shallow hydrothermal system, together with the remaining genes, we assumed one copy per genome.

### Lipid biomarkers and compound specific analysis

#### Lipid extraction

Sediment samples were frozen and freeze dried and then ultrasonically extracted using a mixture of dichloromethane and methanol (DCM:MeOH, 2:1 v/v), repeated 5 times. Extracts were combined and gently evaporated to dryness using a N_2_ blowdown system. The lipid extracts were then re-dissolved and eluted over a glass pipette filled with activated Copper powder to remove sulphur and subsequently split into 3 fractions over a silica column using hexane, hexane/DCM (1:1 v/v) and DCM/MeOH (1:1 v/v). The last eluting fraction was methylated using the method of Ichihara *et al*.^54^ and silylated by adding 20 µl of pyridine and 20 µl of BSTFA (N,O-bis [trimethylsilyl] trifluoroacetamide) and placed in an oven at 60 °C for 25 min.

#### GC/MS analysis

Lipid extracts were analysed using a Shimadzu GCMS-QP2010 Ultra gas chromatography mass spectrometer (GC/MS) with an AOC- 20i auto sampler. Samples were injected in splitless mode onto a Zebron ZB-5HT Inferno GC column (30 m × 0.25 mm × 0.25 μm) using helium as carrier gas. The GC oven temperature was programmed to increase from 60 °C to 180 °C at a ramp of 20 °C min^−1^ followed by a ramp of 4 °C min^−1^ until 320 °C where it was held for 20 min. The total run time was 60 minutes. MS operating conditions were an ion source temperature of 200 °C and 70 eV ionization energy^55^. All peaks were background subtracted and identified by comparing to commercially available or published mass spectra.

#### GC-IRMS analysis

Lipid samples were analysed for δ^13^C values using gas chromatography isotope ratio mass spectrometry (GC-IRMS) using a Thermo Delta V Plus mass spectrometer connected to a Trace Ultra GC, GC Isolink II and Conflo IV. Helium was used as the carrier gas and lipid extracts were injected in PTV mode onto a Zebron ZB-5HT Inferno GC column (30 m × 0.25 mm × 0.25 μm)^56^. The GC oven had an initial temperature of 100 °C then ramping at 20 °C min^−1^ up to 250 °C and 5 °C min^−1^ up to 340 °C, with a hold time of 18 min. Individual compounds were identified by comparing GC-IRMS chromatograms with corresponding GC/MS chromatograms.

### Statistical analysis

Factor analysis^57^ using varimax (variance maximizing) with Kaiser Normalization^58^, was used to quantitatively unravel variability in gene content, between and within the habitats. Factor analysis plot was generated to describe the relationships between genes quantified by qPCR irrespective of the habitat. The 3D factor analysis was made to describe the relationships between each push core and genes quantified by qPCR. Three MANOVA (Multivariate analysis of variance) tested the relationship of the gene quantification between three independent variables: 1) between replicate push cores, 2) habitat, i.e., white- or brown-capped sediments and reference sediments and 3) depth. Multivariate tests of significance were run together with a Tukey’s post hoc tests. The results for which the *P* values are below 0.05 were considered statistically significant. Statistical analyses were performed in SPSS^59^. Principal Component Analysis (PCA) were made using the Raman data in order to find any correlation between habitat and depth. The PCA was performed using XLSTAT^60^, a statistic tool appended to Microsoft Excel.

### Data availability

The datasets generated during and/or analysed during the current study are available from the corresponding author on reasonable request.

## Electronic supplementary material

Supplementary information
