# Supplementary material for: Modes of carbon fixation in an arsenic and CO2-rich shallow hydrothermal ecosystem
Source: Sci Rep. 2017 Oct 31;7:14708. doi: 10.1038/s41598-017-13910-2 (PMC5665909; doi:10.1038/s41598-017-13910-2)
Supplement: Supplementary file 1 — Supplementary information [file 41598_2017_13910_MOESM1_ESM.pdf]

Modes of carbon fixation in an arsenic and CO_2-rich shallow hydrothermal ecosystem

Authors & Affiliations:

Nolwenn Callac[1*], Nicole R. Posth[2], Jayne E. Rattray[1], Kweku. K.Y. Yamoah[1], Alan Wiech[1], Magnus Ivarsson[3], Christoffer Hemmingsson[1], Stephanos P. Kilias[4], Ariadne Argyraki[4], Curt Broman[1], Henrik Skogby[5], Rienk H. Smittenberg[1] and Ernest Chi Fru[1*†]

[1] Stockholm University, Department of Geological Sciences and Bolin Centre for Climate Research, SE-106 91, Stockholm, Sweden.

[2] Nordic Center for Earth Evolution (NordCEE), Department of Biology-University of Southern Denmark Campusvej 55, 5230 Odense M, Denmark.

[3] Department of Palaeobiology and Nordic Center for Earth Evolution, Swedish Museum of Natural History, Stockholm, Sweden.

[4] Department of Economic Geology and Geochemistry Faculty of Geology and Geoenvironment, University of Athens Panepistimiopolis Zographou 157 84 Athens, Greece.

[5] Department of Geosciences, Swedish Museum of Natural History, Stockholm, Sweden.

[†] Present address: School of Earth and Ocean Sciences, Cardiff University, Park Place, Cardiff CF10 3AT, United Kingdom

***Corresponding author:**

Nolwenn.callac@geo.su.se and ChiFruE@cardiff.ac.uk

Supplementary Results

1-Sediment mineralogy and porewater chemistry

The XRD analysis of the white-capped sediments revealed quartz, birnessite (Mn oxides), $(Na,Ca)Mn_7O^{14})_2.8H_2O)$, at all depths. Alunite $(KAl_3(SO_4)_2(OH)_6)$ at 6-8cm (Table S1) was detected in the top and middle depths. In the brown-capped sediments, quartz and birnessite were the main mineral phases at all depths detected by XRD. In the deepest sections, the mica-phases were prevalent (Table S1). In the reference sediments, as is the case for the brown-capped sediments, quartz and birnessite were detected at all depths, in addition to the mica-phase.

The Raman data further reveals graphite as a key component of all the analysed samples, while feldspar, opal, illite and chlorite were detected in most of the samples (Table S2). Hematite was found in all reference and brown-capped sediment samples except in the deepest sandy sample and only in the top layer of the sediment capped by the white deposits (Table S2). Pyrite and marcasite were detected in the deepest white-capped sediments and sulphur in the surface layer, but not elsewhere or in the other samples (Table S2).

Between 3-5 ml of porewater was recovered from most samples and filtered under anoxic conditions in an anaerobic glove bag (Table S3) for chemical analysis. Porewater samples with a volume <1 ml, especially for the white-capped and reference sediments (Table S3), was associated with sediments exhibiting higher porosity. With the exception of the top and the deeper sections of the push core samples from the white deposits, no porewater was recovered for the middle depths after centrifugation (Table S3).

Concentrations of Ca, K and Mn were higher in porewater collected from the white- and brown-capped deposits and lower in reference sediments (Table S3). Total dissolved S, determined as total dissolved sulphur in acidified filtered samples was used as a proxy for sulphate

concentrations[1]. S concentrations showed a larger variability in the brown-capped deposit (7.8×10^2-3×10^6 ppm) compared to the white-capped sediment (3.7×10^3-8.4×10^3 ppm) and sandy reference also had more variable concentrations (0-8.8×10^3 ppm) (Table S3). Low concentrations of porewater Fe were measured; 94, 209 and 946 ppb for the white-capped, brown-capped and reference sediments, respectively.

2-Statistical analysis

The MANOVA test indicates that the 3 habitats: white-, brown-capped and reference sediments, generally plays an important role in genetic abundance ($P<0.005$). However, there is no obvious difference between archaeal 16S rRNA gene and *aclB* gene abundance in the brown-capped and reference sediments (Table S6). The same was observed for the *dsrA* gene distribution between the white and the brown-capped sediments (Table S6). Considering the specific gene abundance and the individual push cores, the MANOVA test pointed out that replicate cores collected in the white-capped sediments show no statistically supported differences. However, the MANOVA test showed that for several of the quantified genes: *Zetaproteobacteria* 16S rRNA, *dsrA*, *aclB*, *cbbL* and *cbbM*, the habitat and the replicate cores are a significant factor controlling gene abundance (Table S7). Also, the *dsrA* gene abundance does not vary with replicate samples between the three habitats. Related to gene abundances and depth, a global transition can be observed in between 4-8 cm, with the exception of the RuBisCO encoded genes (Table S8). Generally, before and after this transition, most of the genes show no significant depth-dependent abundance.

Significant correlations are found between *Zetaproteobacteria* abundance and the *cbbM* genes in all cores ($R^2 > 0.90$), except in the case of push core 2 collected in the brown-capped

sediments. A strong correlation ($R^2 > 0.93$) between the archaeal 16S rRNA gene abundance and the *aclB* gene is also established in the brown-capped sediments. The *pmoA* and *mmoX* gene abundances, key indicators of the aerobic methanotrophy, as well as the anaerobic methane cycle *mcrA* genes, were quantified in all habitats and at most depths (Fig. 4). The sulphate-reducing bacteria *dsrA* gene was also detected in all habitats and depths (Fig. 4). Between the *dsrA* and *mcrA* genes, a significant correlation was found for the white-capped and reference sediments, with R^2=0.98 and 0.69, respectively. In contrast, no correlation ($R^2 = 0$) is observed between the *dsrA* and *mcrA* genes in the brown-capped sediments. With the exception of push core 2 collected in the white-capped sediments, all push cores showed a significant correlation between the *mmoX*, *pmoA* and *mcrA* genes, R^2> 0.73 and R^2> 0.90, respectively. With the exception of the *cbbM* and the *Zetaproteobacteria* 16S rRNA gene abundances, the highest genetic abundance is in the reference sediments and lowest in the white-capped sediments.

Supplementary Methods

1- Sediment mineralogy and porewater chemistry

The Raman data acquisition was obtained with an 1800 lines/mm grating and the excitation was done with an Ar-ion laser (514 nm) source. A confocal Olympus BX41 microscope was combined to the instrument. The laser beam, with a power at the sample surface at 8 mW, was focused through an 80x objective with a long-working distance of 8mm. The analysed spot size was around 1 µm. The spectral resolution was ~0.4 per cm/pixel and the accuracy of the instrument was controlled by repeated use of a silicon wafer calibration standard. The data collection and spectral baseline correction were done with the LabSpec 5 software.

At the Stockholm University, the porewater from the geomicrobiology push cores was extracted anaerobically. The sediments samples were centrifuged 10 min at 6 000rpm and then the porewater was recovered, filtered through 0.2 µm (Filtropur S, Sarstedt) membrane and stored at 4°C. For several sediment samples, porewater quantities were low. Samples from which <1 ml porewater was collected were analyzed without filtration. The seawater overlying the top of the geomicrobiology push core was also analyzed. Prior to elemental analysis, samples were acidified to 0.28 mol L^{-1} with ultra-pure grade HNO_3 and further diluted 100-fold with 0.28 mol L^{-1} HNO_3.

2-Sediment carbon isotope analysis for the assessment of $\delta^{13}C_{DIC}$ and $\delta^{13}C_{POC}$

Samples were collected for $\delta^{13}C_{DIC}$ analysis by extracting sediment porewater with rhizones every 2 cm into 12 mL, vacated and acid-washed exetainers (LabCo, UK) making sure that no headspace remained. The exetainers were capped and stored upside down in the dark at 4°C until analysis. In the lab, the standards were prepared with international (IAEA NBS-18-Calcite (value = -5.014‰$_{VPDB}$ ±0.035) and IAEA-LSVEC Lithium Carbonate (value = -46.6‰ ± 0.2) (Torres et al., 2005) and in-house bicarbonate standards. Here, approximately 0.5 mg of each international standard was weighed into an acid washed exetainer. The exetainer was capped and then flushed with He. Thereafter, 50 µL of 85% phosphoric acid was added with a gas-tight (Hamilton) syringe. The bicarbonate standard was prepared by creating solutions covering the range of expected concentrations in the samples (0-20 mM). As with the international standards, 50 µL of 85% phosphoric acid was added to the samples with a (Hamilton) syringe. Then, 50-300 µL of the standard (sample amount as was added to the samples) were added to the exetainers using a different (Hamilton) syringe. All standards were then allowed to equilibrate for 24 hours before analysis. The samples were prepared by adding 50 µL of 85% phosphoric acid to He-flushed, acid

washed exetainers using a gas-tight (Hamilton) syringe. Then, 50-300 µL of sample were added using a different (Hamilton) syringe, depending on final concentration needed for detection. The exetainers were allowed to equilibrate for 24 hours at room temperature. The samples were analyzed with a GasBench II coupled to a Delta V Plus mass spectrometer (ThermoFinnigan) following the set-up described in Torres et al., 2005[2] with the exception that we did not use a chilled autosampler and compared to international standards (IAEA NBS-18-Calcite (value = -5.014‰$_{VPDB}$ ±0.035) and IAEA-LSVEC Lithium Carbonate (value = -46.6‰ ± 0.2) (Torres et al., 2005). For all carbon isotope analysis, the results are presented in δ notation, such that: $\delta^{13}C = ([^{13}C/^{12}C_{sample}]/[^{13}C/^{12}C_{VPDB}]) - 1 \times 1000$ and referenced against Vienna Pee-Dee Belemnite (SD of repeated measurements yields $\delta^{13}C_{DIC}$ = 0.25-0.29‰).

For sediment $\delta^{13}C_{POC}$, sediment cores were sliced into sections under N_2 atmosphere using an anaerobic glove bag (Glove Bag Inflatable Glove Chamber, Cole-Parmer) at the field station, stored in 50 mL Falcon tubes and immediately frozen until analysis. In the lab, the samples were freeze-dried and acid-treated with 0.2 M HCl to remove any carbonates. The dried samples were then homogenized with a mortar and pestle, weighed into tin cups, and analyzed on an EA-IRMS (Thermo–Delta V Advantage Isotope Ratio MS, EA Flash 2000 Organic elemental analyzer, SDU Odense). The samples were compared to IVA standard Protein ($\delta^{13}C_{VPDB}$ = -26.98 and IVA standard Urea $\delta^{13}C_{VPDB}$ = -45.38) ($\delta^{13}C_{POC}$ SD of repeated measurements in sediment samples = 0.67‰).

Supplementary Information

The inherent bias of the qPCR:

The sensitivity and specificity of qPCR makes it a valuable screening and quantitative tool, but also introduces biases through inefficient nucleic acid extraction, poor purity, and yield[3-7]. Primers set specificity and qPCR parameters are also critical[8,9]. None of the commonly used nucleic acid extraction protocols are universally perfect. Consequently, different techniques may yield different results[3,5]. We used the PowerSoil DNA extraction kit (MoBio), often employed in DNA extraction from marine hydrothermal systems[7,10]. The universal primers used, were designed from gene bank database sequences, consequently we cannot exclude the possibility of lower coverage of 16S rRNA gene abundances, if our samples contained native lineages not having representative sequences in the gene bank and thereby not recognized by the universal primers[11]. For example, universal 16S rRNA gene primers failed to detect anammox lineages in the *Plamctomycetes* identified with specific primers in deep-sea hydrothermal mats[12]. Similar observations were also found in studies using universal 18S rRNA gene and specific *Botryococcus sp.* qPCR primers, in which the specific primers revealed higher abundance of the specific taxa than the total Eukarya[13]. The case of the *Zetaproteobacteria* 16S rRNA specific and total bacteria 16S rRNA gene quantification is most obvious in our data (Fig. 5), which may also be further coupled to gene copy number variability. Moreover, because we compare qPCR data treated with the same methods, we minimize sample treatment biases, enabling comparative analysis across all habitats.

The six main pathways of the inorganic carbon fixation:

The Calvin cycle—the most studied carbon fixation pathway—is found in many photosynthetic and chemolithotrophic prokaryotes, algae and plants[14,15]. Within the *Prokaryotes*, the Calvin cycle is known to be present in *Alpha-*, *Beta-*, *Gamma-, and Zeta-proteobacteria*, the *Cyanobacteria* and as well as in some Firmicutes, Chloroflexi and archaea[14,16-18]. RuBisCO, the main enzyme used for ribulose 1,5-bisphosphate (RuBP) carboxylation in the first critical step of inorganic carbon fixation to organic carbon, is a marker of the Calvin cycle[14,16]. RuBisCO is currently distinguished into forms I, II, III and IV[16,19]. Only forms I-III have RuBP-dependent CO_2 fixing activity, while form IV encodes RuBisCO-like-proteins unable to fix CO_2, but apparently involved in sulphur metabolisms[14,19-21]. In deep and shallow seafloor hydrothermal systems, the *cbbL* and *cbbM* genes encoding the large unit of RuBisCO forms I and II, respectively, have been detected[22-26]. CO_2 fixation by pure fresh water strains (e.g., *Chlorobium* sp., *Rhodospirillim rubrum* and *Rhodopseudomonas capsulate*) the Calvin cycle produces a $\delta^{13}C_{org}$ in the range of -20‰ to -30‰[27].

The rTCA cycle is most effective in anaerobes and microaerophiles and has been detected in the green phototrophic sulfur bacteria and deeply rooted bacterial lineages in phylum *Aquificae*[14,15,20]. The enzyme, ATP citrate lyase (*aclB,* which we quantified in this study) is central to this pathway[15,28] and CO_2 fixation results in $\delta^{13}C_{org}$ values of up to -12‰[14]. The 3-hydroxypropionate Bi-cycle has only been found in the ubiquitous family, Chloroflexaceae, generating $\delta^{13}C_{org}$ values of up to -13.7‰[14]; while the dicarboxylate/4-hydroxybutyrate cycle reported in the anaerobic and microaerophilic Crenarchaeota in the order Thermoproteales and Desulfurococcales and the 3-hydroxypropionate/4-hydroxybutyrate cycle found in aerobic autotrophic members of the Crenarchaeota, as well as the strict anaerobe, *Stygiolobus azoricus*[15],

produce small $\delta^{13}C_{org}$ values of up to -3.8‰[14,15]. Finally, the reductive acetyl-CoA pathway (Wood-Ljungdahl pathway), widespread in acetogenic bacteria and methanogenic archaea, characteristically produces $\delta^{13}C_{org}$ values >-30‰[14,15]. Biological methane cycling by microorganisms in this pathway may contribute to carbon cycling and to CO_2 production through anaerobic and aerobic methanotrophy in various submarine hydrothermal ecosystems.

Both methanogenesis and anaerobic methanotrophy are carried out by members of the domain archaea[29]. However, there is a paucity of direct $\delta^{13}C_{org}$ signature linked to the anaerobic methanotrophy because even some ANME are also able to live alone[30,31], while some live often in consortia with sulfate-reducers, iron-reducers or nitrate-reducers[32], and so far, no pure strain has been isolated. Aerobic methanotrophy is widespread in some *Gammaproteobacteria* and *Alphaproteobacteria*[33,34] and the first cited class is believed to predominate hydrothermal ecosystems[35-37]. While anaerobic methane oxidation is known to be widespread in sedimentary hydrothermal systems[10,38], it is rarely detected in hydrothermal chimneys[39]. The $\delta^{13}C_{org}$ signature of the various methanotrophic processes varies dependent on the source of methane (thermogenic, abiogenic or biogenic). Furthermore, the ribulose monophosphate aerobic methane oxidation pathway in the *Gammaproteobacteria* and mostly the serine pathway for the *Alphaproteobacteria* members[33,34], fractionate carbon differently[40,41].

Supplementary tables:

Supplementary Table 1. Minerals identified by (X-ray diffraction) XRD in various sediment types and the depth.
Yellow represents minerals found in all the habitats and depth. Purple represents mineral detected only in the white-capped sediments. Green represents minerals identified in the in the brown-capped sediments and the sandy reference sediment.

	**Sediments covered by the white deposit**			**Sediments covered by the brown deposit**			**Sandy sediments (reference)**		
	**Top**	**Middle**	**Bottom**	**Top**	**Middle**	**Bottom**	**Top**	**Middle**	**Bottom**
	0-2 cm	6-8 cm	14-16 cm	0-2 cm	6-8 cm	18-20 cm	0-2 cm	6-8 cm	12-14 cm
**Quartz**	X	X	X	X	X	X	X	X	X
**Birnessite**	X	X	X	X	X	X	X	X	X
**Alunite**	X	X							
**Mica-phase**					X	X	X	X	X

Supplementary Table 2: Minerals identified by Raman Spectroscopy in various sediment types and depth.
Yellow represents minerals found in all the habitats and most depths. Purple represents minerals detected only in the white-capped sediments. Green, represent minerals found detected in the brown-capped sediments and the sandy reference sediment.

	**Sediments covered by the white deposit**				**Sediments covered by the brown deposit**				**Sandy sediments (reference)**			
	**W2-S1**	**W2-S3**	**W2-S4**	**W2-S8**	**B2-S1**	**B2-S3**	**B2-S4**	**B2-S10**	**S1-S1**	**S1-S3**	**S1-S4**	**S1-S7**
	0-2 cm	4-6 cm	6-8 cm	14-16 cm	0-2 cm	4-6 cm	6-8 cm	18-20 cm	0-2 cm	4-6 cm	6-8 cm	12-14 cm
**Quartz**	X	X	X	X	X	X	X	X	X	X	X	X
**Chlorite**	X		X	X			X	X	X	X	X	
**Graphite**	X	X	X	X	X	X	X	X	X	X	X	X
**Feldspar**	X		X	X		X			X	X	X	
**Muscovite/Illite**		X		X	X	X	X	X		X	X	X
**Opal**		X	X		X	X	X	X		X	X	X
**Hematite**	X				X	X	X	X	X	X	X	
**Goethite**							X		X	X		X
**Pyrite/marcasite**		X	X	X								
**Sulphur**	X											
**Alunite**						X			X			X
**Heulandite**										X		
**Laumontite**		X										
**Anatase**				X	X							

Supplementary Table 3. Chemical composition of sediment pore water according to sediment type and depth. F is for filtrate of filtered sample, NF for non-filtered sample and <DL for below the detection limit. W, B, R are the white-capped, brown-capped, sandy reference sediments, respectively. The corresponding numbers 1 and 2 are identities of push core 1 and 2. S is the section number. SW stands for seawater collected at the top of the sediment. The detection limit, in ppb are 0.01 for the Ca, Mg; 0.3 for Mn; 0.5 for Cd, Cr, Fe, K, Na; 0.7 for Cu; 2 for As and P and 5 for S.

Sample	Depth in cm	As ppb	Ca ppm	Cd ppb	Cr ppb	Cu ppb	Fe ppm	K ppm	Mg ppm	Mn Ppm	Na ppm	P ppm	S ppm
W1-S1-1 NF	0-2	18681.95	655.98	287.67	837.26	425.06	209.09	460.29	1719.39	3.73	942.18	17.50	8141.78
W2-S1	0-2	501.40	522.93	<DL	<DL	19.44	0	387.87	892.63	2.51	541.35	<DL	3725.29
W2-S8	14-16	42663.5	2284.95	1341.09	<DL	<DL	0	2484.32	1733.45	21.20	119.81	<DL	8419.03
BB1	12-16	155.69	1547.61	<DL	453.50	301.54	94.33	2.34E+06	167.67	3.76	834.03	<DL	952.65
BB2	4-8	167.26	1395515	<DL	356.59	281.71	56.76	2.22E+06	145.07	3.77	828.24	<DL	784.70
B2-S2	2-4	0	325.04	<DL	<DL	<DL	0	261.02	974.05	0.43	770.08	<DL	4843.43
B2-S3	4-6	<DL	507.51	<DL	<DL	4.12	0	487.49	1355.40	1.18	1065.47	<DL	6068.81
B2-S4	6-8	<DL	716.74	<DL	<DL	0	0	1.29E+06	781.21	1.98	915.65	<DL	4354.12
B2-S5	8-10	20.05	1127.83	<DL	<DL	6.27	0	1.3E+06	269.78	1.76	703.77	<DL	2992817
B2-S6	10-12	13.22	1177.36	<DL	<DL	0	0	1.66E+06	263.48	0.09	1143.89	<DL	2001.81
B2-S7	12-14	0	1148.99	<DL	<DL	5.37	0.28	1.76E+06	258.81	0.36	1191.7	<DL	1507.74
B2-S8	14-16	<DL	1143.43	<DL	<DL	0.30	0	1.64E+06	234.16	<DL	1210.3	<DL	179050
B2-S9	16-18	<DL	1355.55	<DL	<DL	0	2.51	1.81E+06	306.95	0.058	1311.18	<DL	1654.36
B2-S10 NF	18-20	150.37	1412.73	<DL	1521.59	651.95	30.66	2.13E+06	493.35	5.07	1589.34	<DL	1576.94
R1-S3	4-6	0	282.24	<DL	<DL	0	0	197.67	889.83	<DL	1094.6	<DL	5273.07
R1-S4 NF	6-8	596.61	741.26	<DL	3279.09	870.05	946.77	673.89	2540.07	13.38	1063.31	<DL	8819.43
R1-S5	8-10	0	270.53	<DL	<DL	0	0	178.53	731.63	<DL	1192.36	<DL	5053.48
R1-S6	10-12	<DL	391.76	<DL	<DL	<DL	0	309.03	1298.99	<DL	1776.1	<DL	5426.03
R2-S1 NF	0-2	243.78	350.27	<DL	738.28	213.58	171.81	259.77	1092.91	2.76	1.64	6.58	4325.46
R2-S2 NF	2-4	125.27	304.16	<DL	459.21	227.08	124.18	239.22	976.44	1.90	1304.08	<DL	4270.34
R2-S7-1	12-16	8.81	0	<DL	<DL	0	0	0	0	<DL	0	<DL	0

R2-S7 NF	12-16	<DL	263.99	<DL	70.90402	12.49	20.83	222.29	908.99	0.32	1413.33	<DL	3487.71
SW-W push core 1 F	Top core	112.08	293.58	<DL	<DL	0	0	217.66	1059.91	0.07	1563.03	<DL	3538.13
SW-W push core 2F	Top core	<DL	168.99	<DL	<DL	0	0	151.93	485.84	0.57	939.57	<DL	2872.77
SW-R push core 1F	Top core	<DL	307.32	<DL	<DL	<DL	0	231.56	1083.31	<DL	1291.39	<DL	3380.87
SW-R push core 2F	Top core	0	170.67	<DL	<DL	0	0	123.09	598.68	<DL	917.24	<DL	3192.78
SW-B push core 1 F	Top core	0	346.77	<DL	<DL	30.38	0	290.13	1166.13	0.14	1075.37	<DL	3234.24

Supplementary Table 4. Relative bacteria and archaea abundance expressed as percentage of total prokaryotes. The total prokaryotes abundance was calculated from quantified archaeal and bacterial 16S rRNA gene abundance.

Depth (cm)	Bacteria (%)	Archaea (%)	Depth (cm)	Bacteria (%)	Archaea (%)	Depth (cm)	Bacteria (%)	Archaea (%)
**White-capped sediment**			**Brown-capped sediment**			**Reference sediment**		
**Push core 1**			**Push core 1**			**Push core 1**		
0-2 cm	95.9	4.1	0-4 cm	98.2	1.8	0-2 cm	96.9	3.1
2-4 cm	67.3	32.7	4-8 cm	95.6	4.4	2-4 cm	99.3	0.7
4-6 cm	33.1	66.9	8-12 cm	83.5	16.5	4-6 cm	99.3	0.7
6-8 cm	38.3	61.7	12 -16 cm	79.9	20.1	6-8 cm	98.3	1.7
8-10 cm	46.4	53.6	**Push core 2**			8-10 cm	98.5	1.5
10-12 cm	46.5	53.5	0-2 cm	98.4	1.6	10-12 cm	98.8	1.2
12-14 cm	55.2	44.8	2-4 cm	98.2	1.8	**Push core 2**		
**Push core 2**			4-6 cm	97.1	2.9	0-2 cm	99.4	0.6
0-2 cm	97.5	2.5	6-8 cm	94.6	5.4	2-4 cm	99.4	0.6
2-4 cm	40.0	60.0	8-10 cm	88.7	11.3	4-6 cm	98.8	1.2
4-6 cm	50.3	49.7	10-12 cm	87.7	12.3	6-8 cm	97.9	2.1
6-8 cm	50.4	49.6	12-14 cm	89.9	10.1	8-10 cm	98.8	1.2
8-10 cm	20.7	79.3	14-16 cm	90.5	9.5	10-12 cm	96.7	3.3
10-12 cm	86.3	13.7	16-18 cm	90.4	9.6	12-14 cm	95.1	4.9
12-14 cm	78.5	21.5	18-20 cm	96.5	3.5			
14-18 cm	13.8	86.2						

Supplementary Table 5: Summary of the most abundant lipid biomarkers from each site in ng g-1 dry weight sediment, concentrations are calculated relative to alkane standards (lod: limit of detection).

	**Sediments covered by the white deposit**			**Sediments covered by the brown deposit**			**Sandy sediments (reference)**		
	**Top** 0-2 cm	**Middle** 6-8 cm	**Bottom** 12-14 cm	**Top** 0-2 cm	**Middle** 6-8 cm	**Bottom** 16-18 cm	**Top** 0-2 cm	**Middle** 6-8 cm	**Bottom** 10-12 cm
**Bacterially derived lipids**									
*anti iso*C15:0 fatty acid	11	<lod	<lod	93	<lod	<lod	256	78	52
*iso*C15:0 fatty acid	44	<lod	<lod	140	<lod	<lod	295	83	112
**Eukaryotically derived lipids**									
Cholesterol Δ^{5}	109	227	34	277	123	<lod	182	133	116
Sterol $\Delta^{5\ 22}$	120	<lod	<lod	123	120	<lod	171	54	142
Stigmasterol $\Delta^{5\ 22}$	76	<lod	<lod	235	175	<lod	300	32	147
Beta sitosterol Δ^{5}	199	<lod	<lod	697	279	<lod	353	58	174
**Lipids of non-specific origin**									
C16:9 fatty acid	112	<lod	<lod	1423	115	<lod	3759	359	415
C16:0 fatty acid	606	435	333	2771	630	233	4055	1256	1035
C18:0 fatty acid	484	423	333	665	821	368	818	707	588

Supplementary Table 6. MANOVA generated P-values for genes quantified by qPCR and sediment type. White, brown, reference and Zeta, represent white-capped, brown-capped, sandy reference sediments and *Zetaproteobacteria*. Values in yellow are significant at $P < 0.05$.

Habitat	Habitat	*Bacteria*	*Archaea*	*mcrA*	*dsrA*	*Zeta*	*pmoA*	*mmoX*	*aclB*	*cbbL*	*cbbM*
"White"	"Brown"	0	0	0	0.538	0	0	0	0	0	0
"White"	"Reference"	0	0	0	0	0	0	0	0	0	0.008
"Brown"	"Reference"	0	0.975	0	0	0	0	0	0.738	0	0

Supplementary Table 7 MANOVA P-values generated genes quantified by qPCR, relative to push core replicates. White PC1 and white PC1, Brown PC1 and Brown PC2, Ref PC1 and Ref PC2 and Zeta are for white-capped, brown-capped, sandy reference push cores and *Zetaproteobacteria*, respectively. Values in yellow are significant at $P<0.05$.

Push core	Push core	*Bacteria*	*Archaea*	*mcrA*	*dsrA*	*Zeta*	*pmoA*	*mmoX*	*aclB*	*cbbL*	*cbbM*
White PC1	White PC2	0.865	0.669	0.356	1	1	0.959	0.959	0.399	0.999	0.992
White PC1	Brown PC1	0	0	0	0.997	0	0	0	0	0	0
White PC1	Brown PC2	0	0	0	0.913	0	0	0	0	0	0
White PC1	Ref PC1	0	0	0	0	0	0	0	0	0	0.43
White PC1	Ref PC2	0	0	0	0	0	0	0	0	0	0.049
White PC2	Brown PC1	0	0	0	0.999	0	0	0.003	0.002	0	0
White PC2	Brown PC2	0	0	0	0.942	0	0	0	0	0	0
White PC2	Ref PC1	0	0	0	0	0	0	0	0	0	0.754
White PC2	Ref PC2	0	0	0	0	0	0	0	0	0	0.164
Brown PC1	Brown PC2	0	0.025	0.026	0.999	0	0.069	0	0	0.302	0
Brown PC1	Ref PC1	0	0.987	0	0	1	0.282	0	0	0	0
Brown PC1	Ref PC2	0	0.01	0	0	0.973	0	0	0.001	0	0
Brown PC2	Ref PC1	0	0.059	0	0	0	0	0	0.814	0	0
Brown PC2	Ref PC2	0	0.998	0	0	0	0	0	0.115	0	0
Ref PC1	Ref PC2	0.001	0.021	0	1	0.975	0	0.002	0.844	0.963	0.944

Supplementary Table 8. MANOVA P-values generated for genes quantified by qPCR relative to sampled depths. Zeta is for *Zetaproteobacteria*. Values in yellow are significant at *P< 0.05*.

Depth (cm)	Depth (cm)	*Bacteria*	*Archaea*	*mcrA*	*dsrA*	*Zeta*	*pmoA*	*mmoX*	*aclB*	*cbbL*	*cbbM*
0-2	2-4	0	0.041	0	0.064	0	0	0.001	0.032	0	0
0-2	4-6	0	0	0	0.849	0	0	0	0	0	0
0-2	6-8	0	0	0	1	0	0.085	0	0	0.994	0
0-2	8-10	0	0	0.112	0.17	0	0	1	0	0.049	0
0-2	10-12	0	0	0	0.277	0	0.197	0	0	0.003	0
0-2	12-14	0	0	0	0.533	0	0	0	0	0	0
0-2	14-16	0	0	0	0.028	0	0	0	0	0	0
2-4	4-6	0	0	0	0	0	0	0	0	0	0
2-4	6-8	0	0	0	0.112	0	0	0	0	0	0
2-4	8-10	0	0	0	1	0	0.006	0	0	0	0
2-4	10-12	0	0	0	0.998	0	0	0	0	0	0
2-4	12-14	0	0	0	0	0	0	0	0	0	0
2-4	14-16	0	0	0	0	0	0	0	0	0	0
4-6	6-8	0.055	0.999	0.005	0.542	0.999	0.283	0.984	0.949	0.001	0.008
4-6	8-10	0	0.933	0	0.003	0.055	0	0	0	0	0.047
4-6	10-12	0.014	1	0.1	0.006	1	0.178	0.004	0.588	0.99	0.001
4-6	12-14	0.592	0.787	0.004	0.999	1	0.834	1	0.74	0.641	0.005
4-6	14-16	0	0	0	0.38	0.53	0	0.056	0.054	0	0.001
6-8	8-10	0	0.996	0	0.28	0.156	0	0	0.01	0.003	1
6-8	10-12	0.999	0.992	0.977	0.434	0.997	1	0.036	0.055	0.016	0.985
6-8	12-14	0	0.956	0	0.241	1	0.009	0.998	0.128	0	0.999
6-8	14-16	0	0	0	0.006	0.222	0	0.005	0.002	0	0.86
8-10	10-12	0	0.825	0	1	0.038	0	0	0	0	0.922
8-10	12-14	0	1	0	0.001	0.092	0	0	0	0	0.988
8-10	14-16	0	0	0	0	0.001	0	0	0	0	0.725
10-12	12-14	0	0.622	0	0.001	1	0.005	0.016	1	0.174	1

10-12	14-16	0	0.001	0	0	0.563	0	0	0.756	0	0.998
12-14	14-16	0.058	0	0.347	0.768	0.562	0.024	0.047	0.753	0.007	0.99

Supplementary Table 9. Primers used for qPCR and representative strains used for standard curve.
cbbL and *cbbM* genes encode the RuBisCO form I and II, respectively in the Calvin cycle; *aclB* encode the ATP citrate lyase β subunit in the rTCA cycle, *pmoA* encode the particulate methane monooxygenase (pMMO) and *mmoX* encoding the soluble methane monooxygenase (sMMO) in the aerobic methane oxidizing Gammaproteobacteria and Alphaproterobacteria, respectively,
mcrA encoding the methyl coenzyme M reductase central enzyme for both methanogenesis and the anaerobic methane oxidation (Hallam et al., 2003; Hallam et al., 2004), *dsrA* encoding dissimilatory sulfite reductase α subunit, a key enzyme for sulfate-reduction

Target (gene)	Strain used for the calibration curve	Primer name	Primer sequence (5' - 3')	Primers concentration (nM)	Annealing temperature (°C)	References
Archaea (16S rRNA)	*Methanoculleus marisnigrii* (DSMZ 1498)	ARC787f	ATTAGATACCCSBGTAGTCC	500	60	[42]
		ARC1059r	GCCATGCACCWCCTCTC	500		
Bacteria (16S rRNA)	*Citreicella thioxydans* (DSMZ 10146)	BACT1369f	CGGTGAATACGTTCYCGG	500	58	[43]
		BACT1492r	GGWTACCTTGTTACGACTT	500		
Iron-oxidizing (16S rRNA)	*Mariprofundus ferrooxydans* (DSMZ 23021)	Zeta672F	CGG AAT TCC GTG TGT AGC AGT	500	60	[44]
		Zeta837R	GCC ACW GYA GGG GTC GAT ACC	500		
cbbL RuBisCO from I	*Synechococcus sp.* (S7942)	cbbLR1F	AAG GAY GAC GAG AAC ATC	500	60	[45]
		cbbLR1int-R	TGC AGS ATC ATG TCR TT	500		
cbbM RuBisCO from II	*Mariprofundus ferrooxydans* (DSMZ 23021)	*cbbM* F	TTC TGG CTG GGB GGH GAY TTY ATY AAR AAY GAC GA	500	60	[46]
		cbbM R	CCG TGR CCR GCV CGR TGG TAR TG	500		
aclB (rTCA cycle)	*Methanoculleus sp.* MAB1	*aclB*892F	TGG ACM ATG GTD GCY GGK GGT	500	60	[47]
		*aclB1204*R	ATA GTT KGG SCC ACC TCT TC	500		
pmoA (pMMO)	*Methanocystis hirsuta* (DSMZ 18500)	A189F	GGN GAC TGG GAC TTC TGG	500	58	[48,49]
		mb661R	CCG GMG CAA CGT CYT TAC C	500		
mmoX (sMMO)	*Methanocystis hirsuta* (DSMZ 18500)	mmoX1	CGG TCC GCT GTG GAA GGG CAT GAA GCG CGT	500	56	[50]
		mmoX2	GGC TCG ACC TTG AAC TTG GAG CCA TAC TCG	500		
mcrA	*Methanoculleus marisnigrii* (DSMZ 1498)	ME3MF	ATGTCNGGTGHGTMGGSTTYA C	500	52	[51,52]
		ME2r'	TCATBGCRTAGTTDGGRTAGT	500		

dsrA	*Desulfovibrio tunisiensis* (DSMZ 19275)	DSR1F+<br>DSR-R	ACS CAC TGG AAG CAC GCC GG<br>GTG GMR CCG TGC AKR TTG G	500<br>500	60	[53]

Supplementary figures:

Supplementary Figure 1. Stable carbon isotope values for sedimentary organic matter ($\delta^{13}C_{org}$) and porewater total dissolved inorganic carbon ($\delta^{13}{}_{DIC}$) for A) white-capped sediments, B) brown-capped sediments, C) reference sediments.

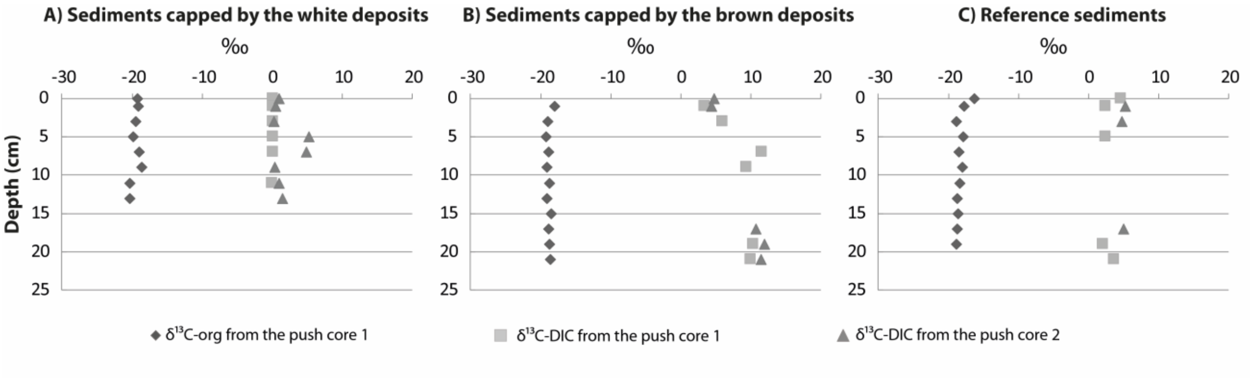

Supplementary Figure 2. Average abundance for each section between the duplicate push cores collected in the same habitat, showing downcore gene distribution. The X-axis refers to the abundance of gene copies per gram of sediment for A) Bacterial 16S rRNA gene. B) Archaeal 16S rRNA gene; C) *Zetaproteobacteria* 16S rRNA gene; D) *aclB* gene; E) the *cbbL* gene; F) the *cbbM* gene. The orange dots refer to qPCR data obtained in the white-capped sediment; the brown dots refer to qPCR data obtained in the brown-capped sediment and the blue dots refer to qPCR data obtained in the reference sediment.

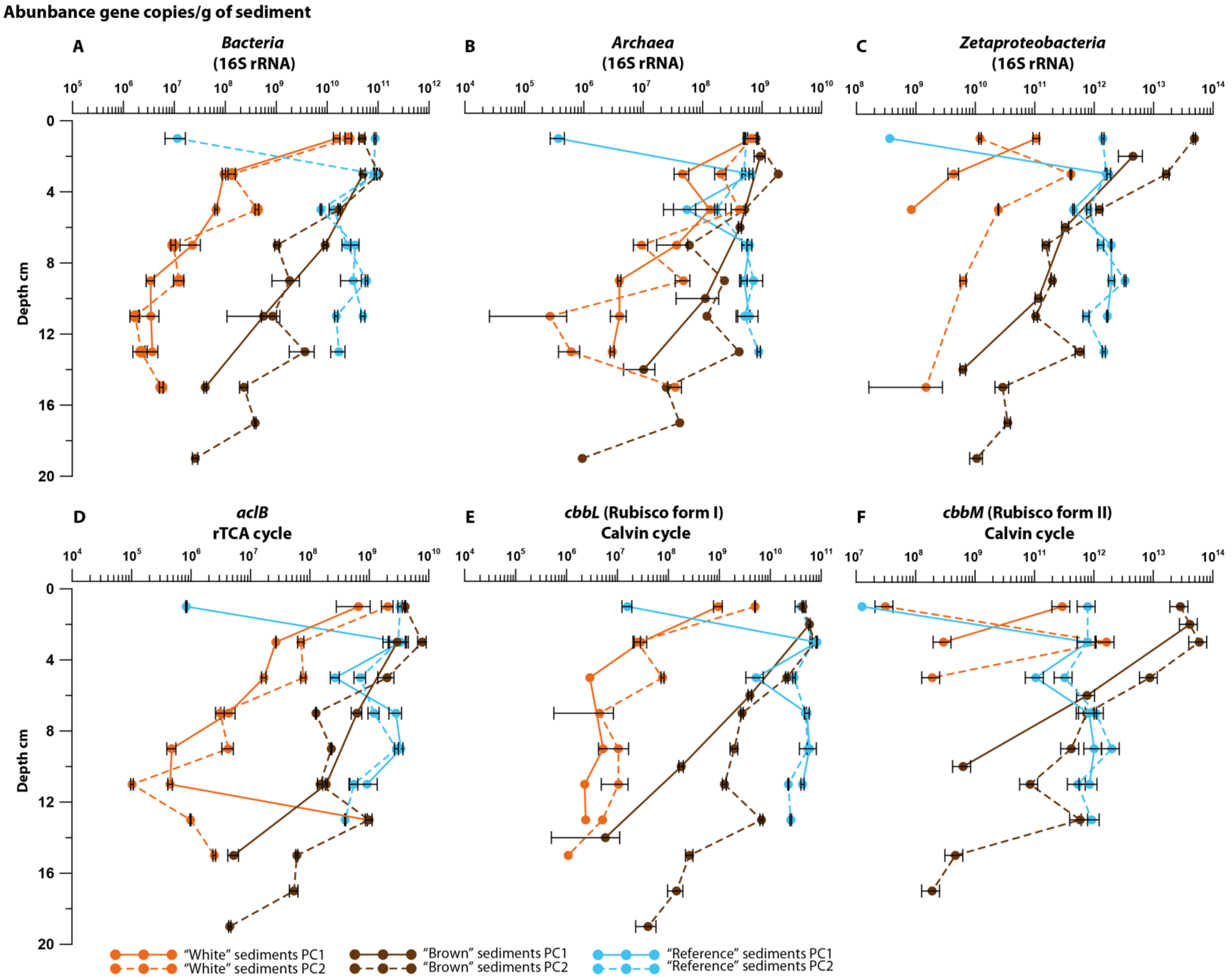
Abunbance gene copies/g of sediment
A
Bacteria (16S rRNA)
B
Archaea (16S rRNA)
C
Zetaproteobacteria (16S rRNA)
D
aclB rTCA cycle
E
cbbL (Rubisco form I) Calvin cycle
F
cbbM (Rubisco form II) Calvin cycle
Depth cm
"White" sediments PC1
"White" sediments PC2
"Brown" sediments PC1
"Brown" sediments PC2
"Reference" sediments PC1
"Reference" sediments PC2

Supplementary Figure 3. Average abundance, according the sediment depth of the gene involved in the methane cycle and in the sulphate-reduction showing downcore gene distribution, for each section between the duplicate push cores collected in the same habitat. The X-axis refers to the abundance of gene copies per gram of sediment for: A) abundance of the *mmoX* genes (encoding the soluble methane monooxygenase, aerobic methane cycle); B) abundance of the archaeal *pmoA* genes (encoding the particulate methane monooxygenase, aerobic methane cycle); C) abundance of the *mcrA* genes (methyl coenzyme M reductase central enzyme for both methanogenesis and the anaerobic methane oxidation) and D) abundance of the *dsrA* genes (sulfate-reduction). The orange dots refer to qPCR data obtained in the white-capped sediment; the brown dots refer to qPCR data obtained in the brown-capped sediment and the blue dots refer to qPCR data obtained in the reference sediment.

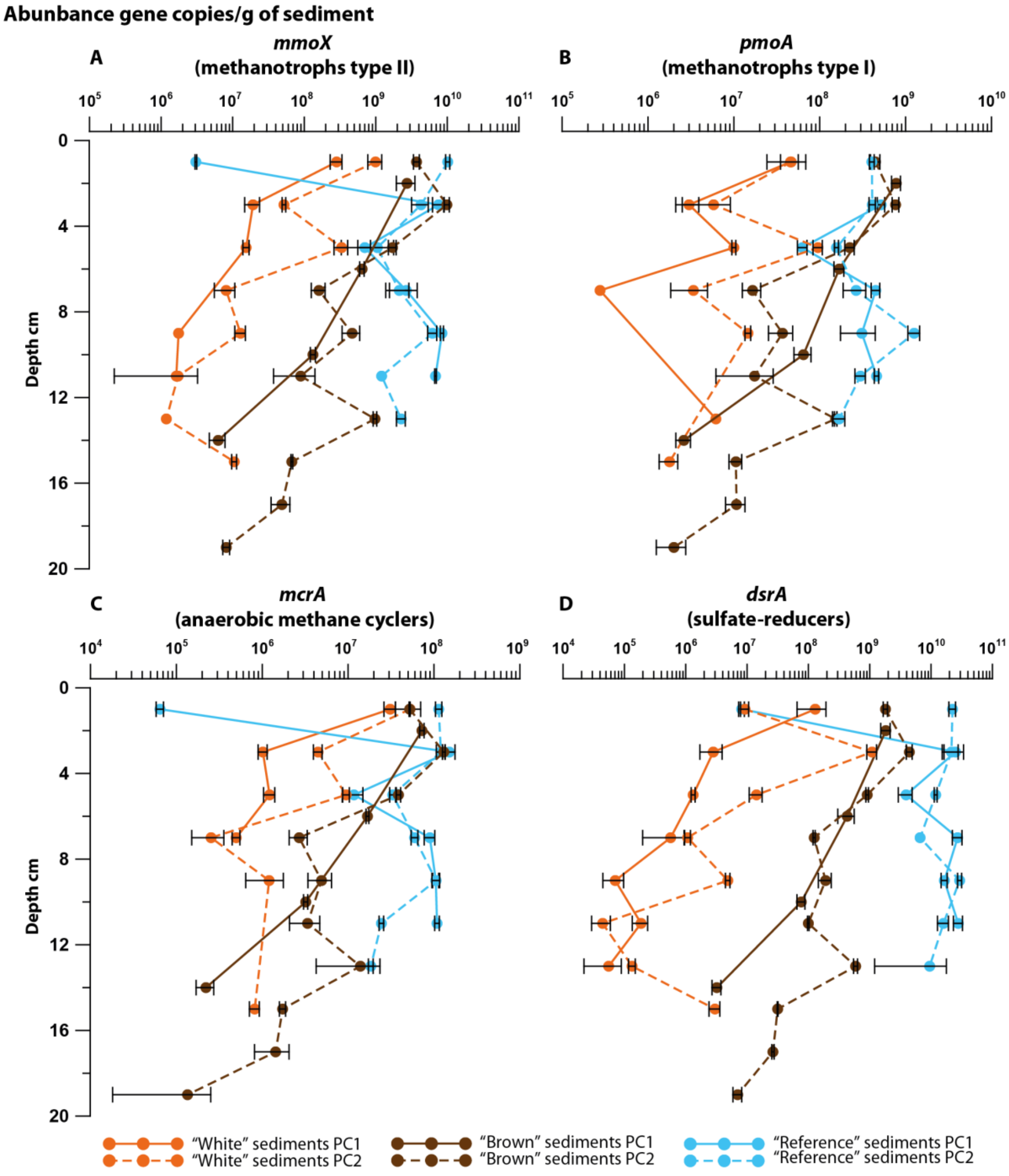

Abunbance gene copies/g of sediment
A
mmoX
(methanotrophs type II)
B
pmoA
(methanotrophs type I)
C
mcrA
(anaerobic methane cyclers)
D
dsrA
(sulfate-reducers)
Depth cm
"White" sediments PC1
"White" sediments PC2
"Brown" sediments PC1
"Brown" sediments PC2
"Reference" sediments PC1
"Reference" sediments PC2

Supplementary Figure 4. Picture showing the seagrass meadow (orange arrows) surrounding our hydrothermal sampling site, 36°40’N; 24°31’E southeast of Milos Island, at 12.5 m below sea level. Bubbling exhibits sign of hydrothermal activity in the white-capped sediment.

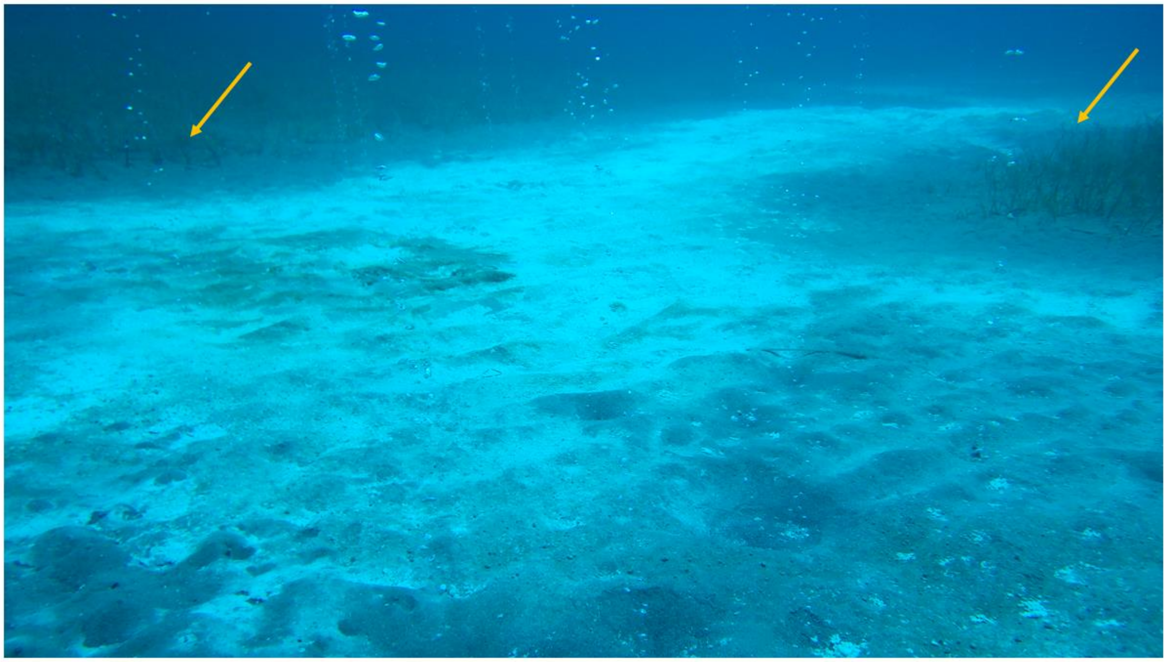

References:

1 Callac, N. *et al.* Microbial colonization of basaltic glasses in hydrothermal organic-rich sediments at Guaymas Basin. *Frontiers in Microbiology* **4**, doi:10.3389/fmicb.2013.00250 (2013).

2 Torres, M. E., Mix, A. C. & Rugh, W. D. Precise δ13C analysis of dissolved inorganic carbon in natural waters using automated headspace sampling and continuous-flow mass spectrometry. *Limnol. Oceanogr.: Methods* **3**, 349-360 (2005).

3 Alain, K. *et al.* DNA extractions from deep subseafloor sediments: Novel cryogenic-mill-based procedure and comparison to existing protocols. *Journal of Microbiological Methods* **87**, 355-362 (2011).

4 Zhou, J., Bruns, M. A. & Tiedje, J. M. DNA recovery from soils of diverse composition. *Applied and Environmental Microbiology* **62**, 316-322 (1996).

5 Webster, G., Newberry, C. J., Fry, J. C. & Weightman, A. J. Assessment of bacterial community structure in the deep sub-seafloor biosphere by 16S rDNA-based techniques: a cautionary tale. *Journal of Microbiological Methods* **55**, 155-164 (2003).

6 Lloyd, K. G., May, M. K., Kevorkian, R. T. & Steen, A. D. Meta-Analysis of Quantification Methods Shows that Archaea and Bacteria Have Similar Abundances in the Subseafloor. *Applied and Environmental Microbiology* **79**, 7790-7799, doi:10.1128/aem.02090-13 (2013).

7 Lloyd, K. G., MacGregor, B. J. & Teske, A. Quantitative PCR methods for RNA and DNA in marine sediments: maximizing yield while overcoming inhibition. *FEMS microbiology ecology* **72**, 143-151 (2010).

8 Wintzingerode, F., Göbel, U. B. & Stackebrandt, E. Determination of microbial diversity in environmental samples: pitfalls of PCR-based rRNA analysis. *FEMS Microbiology Reviews* **21**, 213-229 (1997).

9 Smith, C. J. & Osborn, A. M. Advantages and limitations of quantitative PCR (Q-PCR)-based approaches in microbial ecology. *FEMS microbiology ecology* **67**, 6-20 (2009).

10 Biddle, J. F. *et al.* Anaerobic oxidation of methane at different temperature regimes in Guaymas Basin hydrothermal sediments. *ISME Journal* **6**, 1018-1031, doi:http://www.nature.com/ismej/journal/v6/n5/suppinfo/ismej2011164s1.html (2012).

11 Sievert, S. M., Ziebis, W., Kuever, J. & Sahm, K. Relative abundance of Archaea and Bacteria along a thermal gradient of a shallow-water hydrothermal vent quantified by rRNA slot-blot hybridization. *Microbiology-Uk* **146 Part 6**, 1287-1293 (2000).

12 Crépeau, V. *et al.* Diversity and function in microbial mats from the Lucky Strike hydrothermal vent field. *FEMS Microbiology Ecology* **76**, 524-540, doi:10.1111/j.1574-6941.2011.01070.x (2011).

13 Yamoah, K. A. *et al.* A 150-year record of phytoplankton community succession controlled by hydroclimatic variability in a tropical lake. *Biogeosciences* **13**, 3971-3980, doi:10.5194/bg-13-3971-2016 (2016).

14 Berg, I. A. *et al.* Autotrophic carbon fixation in archaea. *Nature Reviews Microbiology* **8**, 447-460 (2010).

15 Fuchs, G. Alternative pathways of carbon dioxide fixation: insights into the early evolution of life? *Annual review of microbiology* **65**, 631-658 (2011).

16 Tabita, F. R., Satagopan, S., Hanson, T. E., Kreel, N. E. & Scott, S. S. Distinct form I, II, III, and IV Rubisco proteins from the three kingdoms of life provide clues about Rubisco

evolution and structure/function relationships. *Journal of experimental botany* **59**, 1515-1524 (2008).

17 Singer, E. *et al.* Mariprofundus ferrooxydans PV-1 the first genome of a marine Fe (II) oxidizing Zetaproteobacterium. (2011).

18 Elsaied, H. E., Kimura, H. & Naganuma, T. Composition of archaeal, bacterial, and eukaryal RuBisCO genotypes in three Western Pacific arc hydrothermal vent systems. *Extremophiles* **11**, 191-202 (2007).

19 Badger, M. R. & Bek, E. J. Multiple Rubisco forms in proteobacteria: their functional significance in relation to CO2 acquisition by the CBB cycle. *Journal of experimental botany* **59**, 1525-1541 (2008).

20 Saini, R., Kapoor, R., Kumar, R., Siddiqi, T. O. & Kumar, A. CO_2 utilizing microbes — A comprehensive review. *Biotechnology Advances* **29**, 949-960, doi:http://dx.doi.org/10.1016/j.biotechadv.2011.08.009 (2011).

21 Ashida, H., Danchin, A. & Yokota, A. Was photosynthetic RuBisCO recruited by acquisitive evolution from RuBisCO-like proteins involved in sulfur metabolism? *Research in microbiology* **156**, 611-618 (2005).

22 Elsaied, H. & Naganuma, T. Phylogenetic diversity of ribulose-1, 5-bisphosphate carboxylase/oxygenase large-subunit genes from deep-sea microorganisms. *Applied and Environmental Microbiology* **67**, 1751-1765 (2001).

23 Kato, S., Nakawake, M., Ohkuma, M. & Yamagishi, A. Distribution and phylogenetic diversity of cbbM genes encoding RubisCO form II in a deep-sea hydrothermal field revealed by newly designed PCR primers. *Extremophiles* **16**, 277-283 (2012).

24 Tang, K., Liu, K., Jiao, N., Zhang, Y. & Chen, C.-T. A. Functional Metagenomic Investigations of Microbial Communities in a Shallow-Sea Hydrothermal System. *PloS one* **8**, e72958 (2013).

25 Jesser, K. J., Fullerton, H., Hager, K. W. & Moyer, C. L. Quantitative PCR Analysis of Functional Genes in Iron-Rich Microbial Mats at an Active Hydrothermal Vent System (Lō'ihi Seamount, Hawai'i). *Applied and Environmental Microbiology* **81**, 2976-2984 (2015).

26 Oulas, A. *et al.* Metagenomic investigation of the geologically unique Hellenic Volcanic Arc reveals a distinctive ecosystem with unexpected physiology. *Environ. Microbiol.*, doi:10.1111/1462-2920.13095 (2015).

27 Quandt, L., Gottschalk, G., Ziegler, H. & Stichler, W. Isotope discrimination by photosynthetic bacteria. *FEMS Microbiology Letters* **1**, 125-128 (1977).

28 Hugler, M., Huber, H., Molyneaux, S. J., Vetriani, C. & Sievert, S. M. Autotrophic CO2 fixation via the reductive tricarboxylic acid cycle in different lineages within the phylum Aquificae: evidence for two ways of citrate cleavage. *Environmental Microbiology* **9**, 81-92 (2007).

29 Valentine, D. L. Biogeochemistry and microbial ecology of methane oxidation in anoxic environments: a review. *Antonie van Leeuwenhoek* **81**, 271-282 (2002).

30 Milucka, J. *et al.* Zero-valent sulphur is a key intermediate in marine methane oxidation. *Nature* **491**, 541-546 (2012).

31 Scheller, S., Yu, H., Chadwick, G. L., McGlynn, S. E. & Orphan, V. J. Artificial electron acceptors decouple archaeal methane oxidation from sulfate reduction. *Science* **351**, 703-707, doi:10.1126/science.aad7154 (2016).

32 Raghoebarsing, A. A. *et al.* A microbial consortium couples anaerobic methane oxidation to denitrification. *Nature* **440**, 918-921, doi:http://www.nature.com/nature/journal/v440/n7086/suppinfo/nature04617_S1.html (2006).

33 Hanson, R. S. & Hanson, T. E. Methanotrophic bacteria. *Microbiological Reviews* **60**, 439-471 (1996).

34 Semrau, J. D., DiSpirito, A. A. & Yoon, S. Methanotrophs and copper. *FEMS microbiology reviews* **34**, 496-531 (2010).

35 Elsaied, H. E., Hayashi, T. & Naganuma, T. Molecular analysis of deep-sea hydrothermal vent aerobic methanotrophs by targeting genes of 16S rRNA and particulate methane monooxygenase. *Marine biotechnology* **6**, 503-509 (2004).

36 Nercessian, O., Bienvenu, N., Moreira, D., Prieur, D. & Jeanthon, C. Diversity of functional genes of methanogens, methanotrophs and sulfate reducers in deep-sea hydrothermal environments. *Environmental Microbiology* **7**, 118-132 (2005).

37 Guezennec, J. & Fiala-Medioni, A. Bacterial abundance and diversity in the Barbados Trench determined by phospholipids analysis. *FEMS Microbiology Ecology* **19**, 83-93 (1996).

38 Teske, A. *et al.* Microbial Diversity of Hydrothermal Sediments in the Guaymas Basin: Evidence for Anaerobic Methanotrophic Communities. *Applied and Environmental Microbiology* **68**, 1994-2007 (2002).

39 Brazelton, W. J. *et al.* Archaea and bacteria with surprising microdiversity show shifts in dominance over 1,000-year time scales in hydrothermal chimneys. *Proceedings of the National Academy of Sciences* **107**, 1612-1617, doi:10.1073/pnas.0905369107 (2010).

40 Pancost, R. D. & Sinninghe Damsté, J. S. Carbon isotopic compositions of prokaryotic lipids as tracers of carbon cycling in diverse settings. *Chemical Geology* **195**, 29-58, doi:http://dx.doi.org/10.1016/S0009-2541(02)00387-X (2003).

41 Templeton, A. S., Chu, K.-H., Alvarez-Cohen, L. & Conrad, M. E. Variable carbon isotope fractionation expressed by aerobic CH 4-oxidizing bacteria. *Geochimica et Cosmochimica Acta* **70**, 1739-1752 (2006).

42 Yu, Y., Lee, C., Kim, J. & Hwang, S. Group-specific primer and probe sets to detect methanogenic communities using quantitative real-time polymerase chain reaction. *Biotechnology and Bioengineering* **89**, 670-679, doi:10.1002/bit.20347 (2005).

43 Suzuki, M. T., Taylor, L. T. & DeLong, E. F. Quantitative Analysis of Small-Subunit rRNA Genes in Mixed Microbial Populations via 5'-Nuclease Assays. *Applied and Environmental Microbiology* **66**, 4605-4614, doi:10.1128/aem.66.11.4605-4614.2000 (2000).

44 Kato, S., Kobayashi, C., Kakegawa, T. & Yamagishi, A. Microbial communities in iron-silica-rich microbial mats at deep-sea hydrothermal fields of the Southern Mariana Trough. *Environmental Microbiology* **11**, 2094-2111, doi:10.1111/j.1462-2920.2009.01930.x (2009).

45 Selesi, D., Pattis, I., Schmid, M., Kandeler, E. & Hartmann, A. Quantification of bacterial RubisCO genes in soils by *cbbL* targeted real-time PCR. *Journal of microbiological methods* **69**, 497-503 (2007).

46 Campbell, B. J. & Cary, S. C. Abundance of Reverse Tricarboxylic Acid Cycle Genes in Free-Living Microorganisms at Deep-Sea Hydrothermal Vents. *Appl. Environ. Microbiol.* **70**, 6282-6289 (2004).

47 Campbell, B. J., Stein, J. L. & Cary, S. C. Evidence of Chemolithoautotrophy in the Bacterial Community Associated with Alvinella pompejana, a Hydrothermal Vent Polychaete. *Appl. Environ. Microbiol.* **69**, 5070-5078 (2003).

48 Holmes, A. J., Owens, N. J. & Murrell, J. C. Detection of novel marine methanotrophs using phylogenetic and functional gene probes after methane enrichment. *Microbiology* **141**, 1947-1955 (1995).

49 Costello, A. M. & Lidstrom, M. E. Molecular Characterization of Functional and Phylogenetic Genes from Natural Populations of Methanotrophs in Lake Sediments. *Applied and Environmental Microbiology* **65**, 5066-5074 (1999).

50 Miguez, C., Bourque, D., Sealy, J., Greer, C. & Groleau, D. Detection and isolation of methanotrophic bacteria possessing soluble methane monooxygenase (sMMO) genes using the polymerase chain reaction (PCR). *Microbial ecology* **33**, 21-31 (1997).

51 Nunoura, T. *et al.* Quantification of mcrA by fluorescent PCR in methanogenic and methanotrophic microbial communities. *FEMS microbiology ecology* **64**, 240-247 (2008).

52 Hales, B. *et al.* Isolation and identification of methanogen-specific DNA from blanket bog peat by PCR amplification and sequence analysis. *Applied and Environmental Microbiology* **62**, 668-675 (1996).

53 Kondo, R., Shigematsu, K. & Butani, J. Rapid enumeration of sulphate-reducing bacteria from aquatic environments using real-time PCR. *Plankton Benthos Res* **3**, 180-183 (2008).
